# Prevalence of poor sleep quality in COVID-19 patients: a systematic review and meta-analysis

**DOI:** 10.3389/fpsyt.2023.1272812

**Published:** 2024-01-08

**Authors:** Zhen Gui, Yue-Ying Wang, Jia-Xin Li, Xiao-Hong Li, Zhaohui Su, Teris Cheung, Ka-In Lok, Gabor S. Ungvari, Chee H. Ng, Qinge Zhang, Yu-Tao Xiang

**Affiliations:** ^1^Unit of Psychiatry, Department of Public Health and Medicinal Administration, Faculty of Health Sciences, Institute of Translational Medicine, University of Macau, Macao SAR, China; ^2^Centre for Cognitive and Brain Sciences, University of Macau, Macao SAR, China; ^3^Beijing Huilongguan Hospital, Peking University Huilongguan Clinical Medical School, Beijing, China; ^4^School of Public Health, Southeast University, Nanjing, China; ^5^School of Nursing, Hong Kong Polytechnic University, Kowloon, Hong Kong SAR, China; ^6^Faculty of Health Sciences and Sports, Macao Polytechnic University, Macao SAR, China; ^7^Section of Psychiatry, University of Notre Dame Australia, Fremantle, WA, Australia; ^8^Division of Psychiatry, School of Medicine, University of Western Australia, Perth, WA, Australia; ^9^Department of Psychiatry, The Melbourne Clinic and St Vincent's Hospital, University of Melbourne, Richmond, VIC, Australia; ^10^Beijing Key Laboratory of Mental Disorders, National Clinical Research Center for Mental Disorders & National Center for Mental Disorders, Beijing Anding Hospital, Capital Medical University, Beijing, China

**Keywords:** COVID-19, sleep quality, meta-analysis, prevalence, review

## Abstract

**Objective:**

The coronavirus disease (COVID-19) and the public health responses were associated with a huge health burden, which could influence sleep quality. This meta-analysis and systematic review examined the prevalence of poor sleep quality in COVID-19 patients.

**Methods:**

PubMed, Web of Science, Embase, and PsycINFO were systematically searched from their respective inception to October 27, 2022. Prevalence rates of poor sleep were analyzed using a random effects model.

**Results:**

Totally, 24 epidemiological and 12 comparative studies with 8,146 COVID-19 patients and 5,787 healthy controls were included. The pooled prevalence of poor sleep quality based on the included studies was 65.0% (95%CI: 59.56–70.44%, *I*^2^ = 97.6%). COVID-19 patients had a higher risk of poor sleep quality compared to healthy controls (OR = 1.73, 95% CI: 1.30–2.30, *p* < 0.01, *I*^2^ = 78.1%) based on the 12 comparative studies. Subgroup analysis revealed that COVID-19 patients in low-income countries (*p* = 0.011) and in studies using a lower Pittsburgh Sleep Quality Index score cut-off (*p* < 0.001) were more likely to have poor sleep quality. Meta-regression analyses revealed that being female (*p* = 0.044), older (*p* < 0.001) and married (*p* = 0.009) were significantly correlated with a higher risk of poor sleep quality while quality score (*p* = 0.014) were negatively correlated with the prevalence of poor sleep quality in COVID-19 patients.

**Conclusion:**

Poor sleep quality was found to be very common in COVID-19 patients. Considering the negative effects of poor sleep quality on daily life, sleep quality should be routinely assessed and appropriately addressed in COVID-19 patients.

## 1 Introduction

The huge health burden caused by the coronavirus disease (COVID-19) pandemic and the strict public health measures (e.g., social isolation) were associated with adverse physical and mental health outcomes (1, 2) including poor sleep quality (3). For instance, studies found that SARS-CoV-2 may affect negatively sleep in COVID-19 patients via a direct effect on the central nervous system (CNS) (4), or due to other COVID-19 related symptoms, such as fever, headache, dyspnea, myalgia, throat pain, cough, gastrointestinal disturbance, fatigue, anxiety and depression (5, 6), all of which could lead to poor sleep quality (7). A cohort study of hospitalized COVID-19 patients in China showed that 90.2% experienced poor sleep quality based on the Pittsburgh Sleep Quality Index (PSQI) assessment (8).

Poor sleep quality can have a negative impact on health. A study of COVID-19 patients in Turkey showed that poorer sleep quality was associated with a longer duration of hospitalization, while improving sleep quality could significantly reduce the length of hospital and intensive care unit stays (9). In addition, poor sleep quality combined with chronic retinal stimulation by electronic devices and extended or night work shifts, may worsen lung inflammation and aggravate the symptoms of COVID-19 infection (10). Furthermore, poor sleep quality can trigger oxidative stress and antioxidant imbalances and induce health-damaging pro-inflammatory states. To reduce the harmful effects of poor sleep quality on COVID-19 patients and allocate appropriate health resources, it is important to understand its pattern and associated factors (4, 11).

During the past years, prevalence studies of poor sleep quality in COVID-19 patients found wide variations, ranging from 25.5 to 88.6% (12–14), which is probably related to differences in COVID-19 severity and stages (e.g., COVID-19 onset, recovery, acute post-symptom onset), comorbidities, treatment regimens, and measures on sleep quality and cut-off values (15–19). There are various systematic reviews and meta-analyses that focused on the management of COVID-19, such as the role of methylprednisolone (20), aspirin (21), and baricitinib (22) in COVID-19 patients, but no meta-analysis on the prevalence of poor sleep quality in COVID-19 patients has been published. Therefore, we conducted this meta-analysis on the global prevalence of poor sleep quality in COVID-19 patients and its associated factors.

## 2 Methods

### 2.1 Search strategy

This meta-analysis was conducted based on the Preferred Reporting Items for Systematic Review and Meta-Analyses (PRISMA) (23) and Meta-analysis of Observational Studies in Epidemiology (MOOSE) recommendations (24). The protocol was registered in the International Platform of Registered Systematic Review and Meta-analysis Protocols (INPLASY), with the registration number of INPLASY202320121. Three researchers (ZG, YY-W, and JX-L) independently retrieved relevant literature in PubMed, Web of Science, Embase, and PsycINFO databases from their inception until October 27, 2022, with the following search terms: (SARS-CoV-2 [MeSH] OR SARS Coronavirus 2 OR Coronavirus 2, SARS OR Coronavirus Disease 2019 Virus OR 2019 Novel Coronavirus OR 2019 Novel Coronaviruses OR Coronavirus, 2019 Novel OR Novel Coronavirus, 2019 OR SARS-CoV-2 Virus OR SARS CoV 2 Virus OR SARS-CoV-2 Viruses OR Virus, SARS-CoV-2 OR 2019-nCoV OR COVID-19 Virus OR COVID 19 Virus OR COVID-19 Viruses OR Virus, COVID-19 OR COVID19 Virus OR COVID19 Viruses OR Virus, COVID19 OR Viruses, COVID19 OR 2019 novel coronavirus infection OR COVID19 OR coronavirus disease 2019 OR coronavirus disease-19 OR 2019-nCoV disease OR 2019 novel coronavirus disease OR 2019-nCoV infection OR COVID-19 patients OR COVID-19 patient OR COVID-19 survivor) AND (Sleep Quality [MeSH] OR Qualities, Sleep OR Quality, Sleep OR Sleep Qualities OR quality of sleep OR sleeping quality OR Pittsburgh sleep quality index OR PSQI).

### 2.2 Study selection

The same researchers independently screened the titles and abstracts of all relevant publications, and then read the full texts to determine eligibility. Any disagreement was resolved by consensus among the three researchers or by discussion with a senior researcher (YT-X). Supplementary Figure S1 shows the detailed process of the literature search. The inclusion criteria for this study were based on the PICOS acronym (25): Participants (*P*): COVID-19 patients based on positive Coronavirus RT-PCR (reverse transcription-polymerase chain reaction) of nasopharyngeal and oropharyngeal swabs or a history of COVID-19 infection. Following previous research (26, 27), the COVID-19 patients in this study included the period of COVID-19 infection, symptom onset, recovery, and the onset of post-acute COVID-19 symptoms. Interventions (*I*): not applicable; Comparisons (*C*): healthy controls in comparative studies, or not applicable to epidemiological surveys; Outcome (*O*): the prevalence of poor sleep quality or available data could yield the prevalence of poor sleep quality in COVID-19 patients. Sleep quality in COVID-19 patients was assessed using any standardized scales such as the PSQI (7, 28); Study design (*S*): epidemiological and comparative studies (only the baseline data of cohort study were extracted). Exclusion criteria were as follows: (1) published in non-English language; (2) studies that only used individual items rather than the full version of a standardized scales on sleep quality; (3) patients with sleep-related disorders (as recommended in a previous meta-analysis) (29). If a dataset was used in multiple papers, only the paper with the complete information was included in this meta-analysis.

### 2.3 Data extraction and quality assessment

The same researchers independently extracted relevant information from the included studies and recorded them using an Excel data collection spreadsheet. The following information was extracted: study characteristics [e.g., title, journal, first author, publication year, study site by country, survey time, study design, assessment method, day zero (26), sampling method, cut-off value of standard scales on sleep quality, study quality score, and comorbidities associated with sleep quality], characteristics of COVID-19 patients [e.g., number of COVID-19 patients, number with poor sleep quality, total scores of standard scales on sleep quality, mean age (year), proportion of males, proportion of married status, proportion of smoking patients, average days of hospitalization, proportion of ICU admission, prevalence of anxiety and depression, and component scores of standard scales on sleep quality (i.e., subjective sleep quality, sleep latency, sleep duration, habitual sleep efficiency, sleep disturbance, use of sleep medications, daytime dysfunction in the PSQI)] and characteristics of healthy controls [e.g., number of healthy controls, number of poor sleep quality, total scores of standard scales on sleep quality, mean age (year), and proportion of males].

For epidemiological studies, study quality was assessed using an eight-item assessment instrument with a scale of 0–8 (30), with scores of 0–3, 4–6, and 7–8 were considered “low quality”, “moderate quality”, and “high quality,” respectively (31). Study quality of comparative studies was assessed using the Newcastle-Ottawa Scale (NOS) (32). The NOS included eight items in three categories. In addition to the maximum of 2 stars for comparability, the remaining items could be rated up to 1 star, with a full score of 9 stars. Therefore, the NOS total score in this study ranges from 1 to 9 points, with a higher score indicating higher quality. Details of the study quality assessment tools are shown in Supplementary Table S1.

### 2.4 Statistical analysis

All data analyses were performed using R software (version 4.2.2^1^) with the “meta” package (33). The random-effects model was used to estimate the pooled prevalence of poor sleep quality with the corresponding 95% confidence intervals (95% CIs). The *I*^2^ statistic was used to assess the heterogeneity of the study, with *I*^2^ greater than 50% indicating high heterogeneity (34). Subgroup analyses were performed based on the following categorical variables if there were at least 3 studies in each subgroup (35): cut-off values of standard scales on sleep quality, income levels by country (i.e., High income, vs. upper middle income, vs. lower middle income) according to the World Bank standard,^2^ assessment method (e.g., online survey vs. face-to-face interview), day zero (e.g., symptom onset vs. hospital discharge vs. hospital admission of COVID-19 patients), study design, gender, and sampling method. If there were at least 10 studies (36), meta-regression analyses were performed for the following continuous variable: sample size, mean age (year), proportion of males, proportion of married status, proportion of smoking patients, average days of hospitalization, proportion of ICU admission, quality score, and the prevalence of anxiety and depression. Funnel plot and Egger’s test were used to examine the publication bias. Sensitivity analysis examined the consistency of preliminary results by excluding studies one by one. Significant level was set at *p* < 0.05 (two-tailed).

## 3 Results

### 3.1 Search results and study characteristics

A total of 4,238 relevant publications were initially searched and finally, 36 studies met the study entry criteria and were included in this meta-analysis, of which there were 24 epidemiological and 12 comparative studies (Supplementary Figure S1). After reading the titles and abstracts initially, 293 articles were screened for eligibility by reading the full text, of which, 256 articles were excluded. The reasons for exclusion are outlined in Supplementary Table S2.

A total of 8,146 COVID-19 patients and 5,787 healthy controls were included in this study and their basic characteristics are shown in Table 1. The age range of the participants were between 18 and 99 years. The most number of studies were conducted in Turkey (6 studies, 16.7%), followed by China (5 studies, 13.9%) and Spain (4 studies, 11.1%). Most of the included studies were cross-sectional (26 studies, 72.2%), followed by cohort studies (6 studies, 19.4%). All studies used the PSQI as the measure on sleep quality; 16 studies used the PSQI cut-off values of ≥6 (44.4%), while 14 studies used the cut-off values of ≥5 (38.9%) and 6 studies used the cut-off values of ≥8 (16.7%). Twenty-one studies reported comorbid anxiety and depression, respectively. The study quality assessment scores of 24 epidemiological studies ranged from 4 to 7, of which only 1 study was rated as “high quality,” and the remaining 23 studies (95.8%) were rated as “moderate quality.” In addition, quality assessment scores of the 12 comparative studies ranged from 4 to 6, with a mean score of 5.1 (SD: 0.8) (Supplementary Table S1).

**Table 1:** Basic characteristics of studies included in this meta-analysis.

No.	First author, Publication year	Study site (Country)	Survey time	Study design	Assessment method	Day zero	Sampling method	PSQI Cut-off	COVID-19 patients											Healthy control					Quality score
									No. of COVID-19 patients (n)	No. of PSQ	PSQI total score (mean)	Mean age (years)	Male (%)	Married (%)	Smoking (%)	Average days at hospitalization	ICU admission (%)	Anxiety (%)	Depression (%)	No. of healthy controls (n)	No. of PSQ	PSQI total score (mean)	Mean age (years)	Male (%)	
1	Abbas et al. (37)	Kuwait	May–July 2020	Cross-sectional	Online survey	Symptom onset	NR	≥6	7	6	NR	NR	NR	NR	NR	NR	NR	NR	NR	210	165	NR	NR	NR	5
2	Abdelghani et al. (38)	Egypt	September–November 2020	Cross-sectional	Clinic interview	Hospital discharge	C	≥5	85	65	NR	36.0	18.8	88.2	NR	NR	NR	25	29	85	39	NR	33.7	27.1	6
3	Ahmed et al. (12)	Egypt	January -Mach 2021	Cross-sectional	Clinic follow up	Hospital discharge	NR	≥5	182	118	NR	46.5	46.2	70.3	30.8	12.2	NR	28	12	NR	NR	NR	NR	NR	4
4	Akinci et al. (9)	Turkey	April–May 2020	Cross-sectional	Hospital survey	Hospital admission	NR	≥5	189	102	NR	46.3	59.0	82.5	NR	7.3	NR	13	43	NR	NR	NR	NR	NR	6
5	Akova et al. (39)	Turkey	March–April 2021	Cross-sectional	Clinic interview	Hospital discharge	NR	≥5	133	79	5.7	36.0	45.1	67.7	26.3	NR	NR	NR	NR	NR	NR	NR	NR	NR	5
6	Al-Ameri et al. (40)	all Arabic nations	January–March 2021	Case–control	Online survey	Hospital discharge	NR	≥6	256	215	8.8	37.1	NR	NR	NR	NR	5.1	NR	NR	491	384	8.1	36.2	NR	6
7	Al-Otaibi et al. (41)	Kuwait	August 2020–April 2021	Cross-sectional	Online survey	Symptom onset	R	≥6	13	10	NR	NR	NR	NR	NR	NR	NR	62	NR	93	59	NR	NR	NR	4
8	Alshumrani et al. (17)	Saudi Arabia	May–September 2020	Cross-sectional	Online survey	Symptom onset	C	≥6	643	425	6.9	42.8	61.6	NR	NR	NR	NR	NR	40	448	326	7.6	37.3	52.0	4
9	Amra et al. (13)	Iran	May–June 2020	Cross-sectional	NR	Hospital discharge	NR	≥5	35	31	9.0	34.4	28.6	77.1	NR	NR	NR	57	63	337	261	6.9	34.5	34.7	5
10	Awan et al. (42)	Pakistan	April–September 2021	Cross-sectional	Hospital follow up	Hospital discharge	NR	≥6	50	32	NR	49.6	52.0	66.0	NR	NR	NR	24	10	NR	NR	NR	NR	NR	4
11	Benitez et al. (43)	Spain	March 2020–April 2021	Cohort	Clinic interview	ICU discharge	C	≥6	172	104	7.1	60.5	67.4	NR	45.9	24.6	100	25	15	NR	NR	NR	NR	NR	4
12	Bungenberg et al. (44)	Germany	August 2020–March 2021	Longitudinal prospective	Interview	Symptom onset	NR	≥6	36	31	9.3	NR	NR	NR	26.0	NR	NR	NR	NR	NR	NR	NR	NR	NR	4
13	Cacciatore et al. (45)	Italy	February–April 2020	Cohort	Clinic interview	Hospital discharge	NR	≥5	83	62	6.5	66.9	75.9	NR	NR	13.0	NR	15	16	NR	NR	NR	NR	NR	5
14	Chhajer et al. (71)	India	June 2021	Cross-sectional	Online survey	Hospital discharge	P	≥5	311	245	8.2	NR	39.5	49.8	NR	NR	NR	31	38	NR	NR	NR	NR	NR	4
15	Choudhry et al. (46)	Pakistan	January–March 2021	Longitudinal	NR	Symptom onset	C	≥5	445	201	6.3	42.0	65.3	NR	22.6	4.0	100	NR	NR	NR	NR	NR	NR	NR	6
16	Dai et al. (47)	China	February 2020	Cross-sectional	Online survey	Hospital admission	NR	≥6	307	260	NR	NR	56.7	81.8	21.8	NR	NR	19	13	NR	NR	NR	NR	NR	5
17	Del Brutto et al. (83)	Ecuador	May–June 2020	Longitudinal prospective	Face-to-face interview	Hospital discharge	NR	≥6	325	184	NR	NR	NR	NR	NR	NR	NR	NR	NR	314	127	NR	NR	NR	6
18	Delgado-Alonso et al. (19)	Spain	NR	Cross-sectional	NR	Post-acute COVID-19 syndrome	NR	≥6	50	40	10.1	51.1	26.0	NR	8.0	19.1	10.0	52	30	NR	NR	NR	NR	NR	5
19	ElHafeez et al. (48)	Egypt	April–June 2020	Cross-sectional	Online survey	Hospital discharge	C	≥6	25	19	NR	NR	NR	NR	NR	NR	NR	NR	NR	975	665	NR	NR	NR	6
20	Fernandez et al. (5)	Spain	January–February 2021; July–August 2021	Cohort	Telephone interview	Hospital discharge	NR	≥8	164	69	5.6	60.7	42.1	NR	NR	16.9	17.7	1	8	NR	NR	NR	NR	NR	4
21	Fernandez et al. (5)	Spain	March–June 2020	Cohort	Telephone interview	Hospital discharge	R	≥8	1969	674	6.5	61.1	52.7	NR	NR	11.2	6.5	15	19	NR	NR	NR	NR	NR	7
22	Gundogdu et al. (49)	Turkey	August 2020–March 2021	Cross-sectional	Clinic interview	Hospital discharge	NR	≥5	83	47	NR	57.0	59.0	NR	36.0	15.3	30.1	NR	NR	NR	NR	NR	NR	NR	5
23	Gunes et al. (50)	Turkey	May–June 2020	Cross-sectional	Interview	Hospital admission	NR	≥6	49	27	6.1	46.0	55.1	89.8	NR	NR	NR	NR	NR	NR	NR	NR	NR	NR	4
24	Hartung et al. (51)	Germany	November 2020–September 2021	Prospective multicenter	Clinic interview	Hospital discharge	R	≥5	634	513	NR	NR	NR	NR	NR	NR	NR	NR	NR	NR	NR	NR	NR	NR	6
25	Henriquez et al. (52)	Chile	April–July 2020	Cross-sectional	Questionnaire survey	Hospital discharge	NR	≥6	60	52	9.9	46.3	53.3	NR	46.7	NR	NR	23	10	NR	NR	NR	NR	NR	4
26	Karaogullarindan et al. (53)	Turkey	February–March 2021	Cross-sectional	Questionnaire survey	Hospital admission	NR	≥5	71	38	6.6	60.0	57.7	NR	NR	NR	NR	66	63	71	23	4.8	53.5	57.7	4
27	Li, X. et al. (14)	China	February–March 2020	Cross-sectional	Online survey	Diagnosis date	NR	≥8	51	13	NR	NR	42.4	86.4	18.0	NR	NR	23	28	NR	NR	NR	NR	NR	6
28	Li, Z. et al. (18)	China	April–May 2021	Cross-sectional	Questionnaire survey	Hospital discharge	NR	≥6	535	252	6.2	50.8	40.4	NR	12.1	NR	NR	16	21	NR	NR	NR	NR	NR	6
29	Lin et al. (54)	China	February 2020	Cross-sectional	Online survey	Hospital discharge	NR	≥8	29	11	NR	NR	NR	NR	NR	NR	NR	NR	NR	1868	558	NR	NR	NR	6
30	Malik et al. (55)	Pakistan	April–September 2020	Cross-sectional	Online survey	Diagnosis date	R	≥6	296	182	7.0	NR	38.2	NR	NR	NR	NR	NR	NR	301	146	5.8	NR	43.9	5
31	Nowakowski et al. (56)	USA	August 2020–September 2021	Cohort	Clinic interview or 1 h long telehealth	Hospital discharge	C	≥5	79	65	9.7	48.2	30.4	NR	NR	NR	11.4	38	47	NR	NR	NR	NR	NR	4
32	Rousseau et al. (15)	Belgium	March–July 2020	Cohort	Face-to-face follow up	ICU discharge	NR	≥5	32	24	6.8	60.3	71.9	NR	NR	40.3	100	25	13	NR	NR	NR	NR	NR	5
33	Sljivo et al. (57)	Bosnia, Herzegovina, Croatia, Serbia	February–August 2021	Cross-sectional	Online survey	Symptom onset	NR	≥5	464	362	7.0	NR	18.1	34.3	NR	NR	NR	NR	NR	594	362	5.4	NR	19.0	5
34	Tanriverdi et al. (58)	Turkey	January–February 2021	Cross-sectional	NR	Hospital discharge	NR	≥6	48	24	6.1	39.2	45.8	72.9	6.3	NR	NR	33	29	NR	NR	NR	NR	NR	4
35	Yadav et al. (61)	India	June–August 2020	Cross-sectional	Hospital survey	Diagnosis date	NR	≥8	100	62	8.8	42.9	73.0	NR	33.0	NR	NR	67	27	NR	NR	NR	NR	NR	5
36	Zhang J et al. (16)	China	January–March 2020	Cohort	Hospital survey	Hospital discharge	NR	≥8	135	75	9.0	63.0	57.8	95.6	3.0	31.3	6.7	NR	NR	NR	NR	NR	NR	NR	5

ICU, Intensive Care Unit; PSQI, Pittsburgh Sleep Quality Index; NR, not reported; C, Convenient; R, Random; P, Purposive; No. of PSQ, Number of poor sleep quality participants.

### 3.2 Pooled prevalence of poor sleep quality, PSQI global and component score in COVID-19 patients

**Figure 1:**
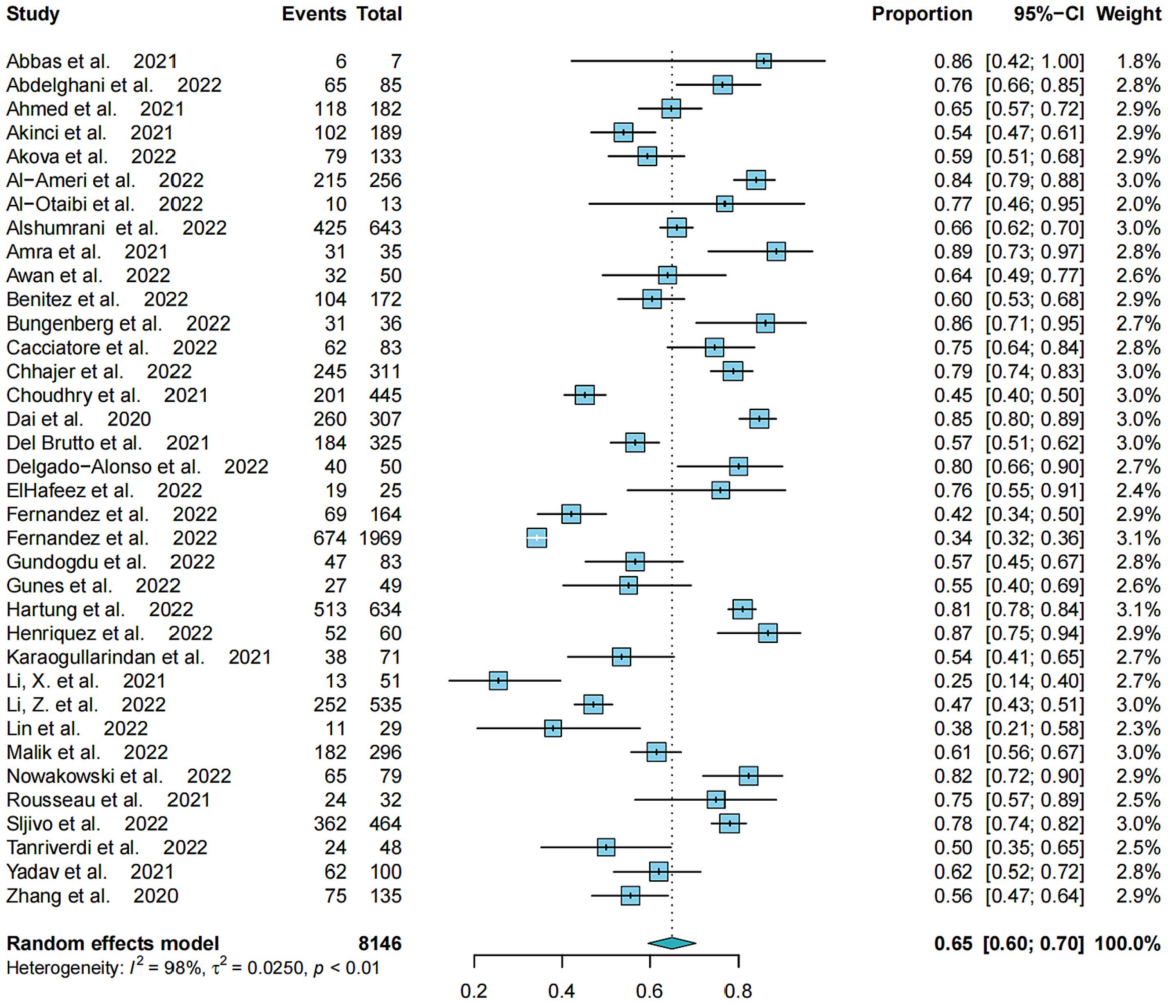
Meta-analysis of prevalence of poor sleep quality in COVID-19 patients.

The pooled prevalence of poor sleep quality based on all 36 studies was 65.0% (95%CI: 59.56–70.44%, *I*^2^ = 97.6%) (Figure 1). Table 2 shows that a total of 21 studies with 5,999 COVID-19 patients reported on the total PSQI score, with a pooled total PSQI score of 7.44 (95% CI: 6.82–8.05, *I*^2^ = 94.2%). In addition, 6 studies with 1,014 COVID-19 patients reported on the sleep component scores in seven domains, with pooled data ranging from 0.53 (95% CI: 0.26–0.79, *I*^2^ = 90.2%) for “use of sleep medications” to 1.50 (95% CI: 1.28–1.73, *I*^2^ = 89.2%) for “sleep latency.”

**Table 2:** The pooled PSQI global and sleep component scores in COVID-19 patients.

Variables	Number of studies	Sample size	Pooled mean score (95% CI)	*I* ^2^	*p* values
PSQI global score	21	5,999	7.44 (6.82–8.05)	94.2%	<0.001
Subjective sleep quality	6	1,014	1.37 (1.12–1.63)	94.6%	<0.001
Sleep latency	6	1,014	1.50 (1.28–1.73)	89.2%	<0.001
Sleep duration	6	1,014	1.12 (0.89–1.36)	92.4%	<0.001
Habitual sleep efficiency	6	1,014	0.84 (0.65–1.02)	84.1%	<0.001
Sleep disturbance	6	1,014	1.32 (1.15–1.50)	93.0%	<0.001
Use of sleep medications	6	1,014	0.53 (0.26–0.79)	90.2%	<0.001
Daytime dysfunction	6	1,014	1.10 (0.88–1.32)	92.6%	<0.001

CI, confidence interval; PSQI, Pittsburgh Sleep Quality Index.

### 3.3 Odds ratio for poor sleep quality between COVID-19 patients and healthy controls

**Figure 2:**
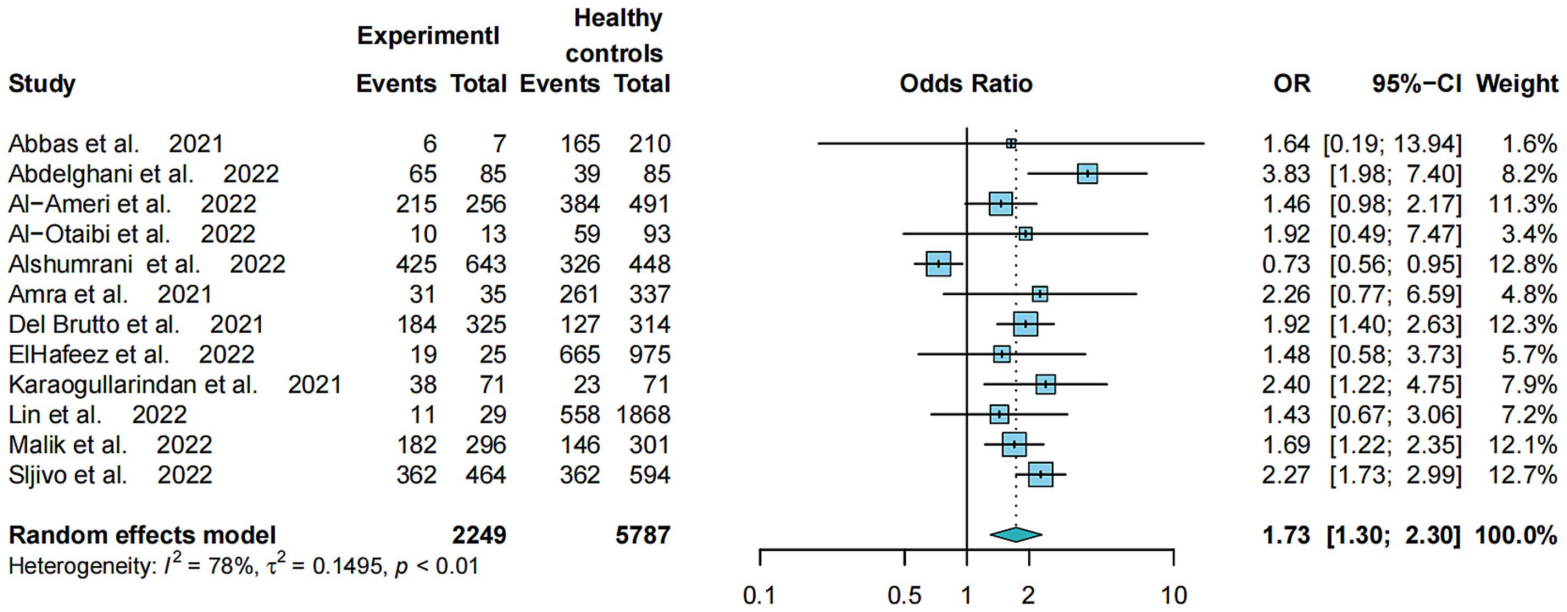
Odds ratio for poor sleep quality between COVID-19 patients and healthy controls.

The Odds Ratio (OR) of the 12 comparative studies on the prevalence of poor sleep quality between COVID-19 patients and healthy controls is shown in Figure 2. Specifically, COVID-19 patients had a higher risk of poor sleep quality compared to healthy controls (OR = 1.73, 95% CI: 1.30–2.30, *p* < 0.01, *I*^2^ = 78.1%). Moreover, COVID-19 patients had significantly higher PSQI total scores than healthy controls, with a large effect size (SMD = 0.31, 95%CI: 0.07–0.55, *p* < 0.0001, *I*^2^ = 92%) (Supplementary Figure S3).

### 3.4 Subgroup and meta-regression analyses

The results of subgroup analysis showed that the cut-offs of PSQI (*p* < 0.001) and countries by income (*p* = 0.011) were significantly associated with the prevalence of poor sleep quality in COVID-19 patients. COVID-19 patients in lower middle-income countries were more likely to have poor sleep quality than those in upper middle income and high-income countries (64.9% vs. 60.1% vs. 55.8%). Meta-regression analyses of 24 epidemiological studies revealed that mean age (β = 1.0000, *z* = 42.6046, *p* < 0.001) and married status (β = 0.0016, *z* = 2.6261, *p* = 0.009) were positively correlated with the prevalence of poor sleep quality in COVID-19 patients, while proportion of males (β = −0.0003, *z* = −2.0108, *p* = 0.044) and quality score (β = −0.0873, *z* = −2.4469, *p* = 0.014) were negatively correlated with the prevalence of poor sleep quality in COVID-19 patients (Table 3).

**Table 3:** Subgroup and meta-regression analyses of prevalence of poor sleep quality in COVID-19 patients.

Subgroup analysis								
Subgroups	No. of studies	No. of studies	Sample size	Having poor sleep quality	Proportion (95% CI)	*I*^2^(%)	*p*-value within subgroups	*p*-value across subgroups
Cut-offs of PSQI	≥5	14	2,826	1,952	69.2 (62.1; 76.2)	94.8	<0.01	<**0.001**
	≥6	16	2,872	1,863	69.5 (62.5; 76.5)	94.7	<0.01	
	≥8	6	2,448	904	43.1 (32.2; 54.1)	91.3	<0.01	
Countries by income	High income	13	3,942	2,075	55.8 (55.4; 57.2)	98.6	<0.01	**0.011**
	Upper middle income	12	1,955	1,112	60.1 (58.1; 62.2)	95.2	<0.01	
	Lower middle income	9	1,529	955	64.9 (62.6; 67.2)	94.1	<0.01	
Assessment method	Online survey	11	2,402	1,748	74.7 (73.0; 76.4)	94.2	<0.01	0.474
	Interview	16	4,265	2,196	54.1 (52.7; 55.4)	98.1	<0.01	
Day zero	Symptom onset	6	1,608	1,035	71.5 (58.1; 84.8)	96.2	<0.01	0.642
	Hospital discharge	22	5,425	2,960	65.2 (58.3; 72.1)	98.1	<0.01	
	Hospital admission	4	616	427	62.5 (46.7; 78.3)	96.1	<0.01	
Study design	Cross-sectional	26	4,016	2,602	65.2 (58.8; 71.6)	94.0	<0.01	0.806
	Cohort	6	2,470	1,004	63.2 (48.7; 77.7)	97.9	<0.01	
Gender	male	4	465	218	46.9 (42.3; 51.4)	0.0	0.53	**0.025**
	female	4	527	290	60.1 (49.4; 70.8)	83.8	<0.01	
Sampling method	Convenient	6	1,449	879	67.1 (55.9; 78.3)	94.5	<0.01	0.713
	Random	4	2,912	1,379	62.6 (41.1; 84.0)	99.5	<0.01	
**Meta-regression Analysis in 24 epidemiological studies**								
**Variables**	**Number of studies**	**Coefficient**		**Standard error**	**95% lower**	**95% Upper**	***Z*-value**	***p*-value**
Sample size	24	−0.0001		0.0002	−0.0006	0.0003	−0.5559	0.578
Mean age (year)	16	1.0000		0.0235	0.9540	1.0460	42.6046	**<0.001**
Male gender, %	22	−0.0003		0.0002	−0.0006	−0.0000	−2.0108	**0.044**
Married, %	10	0.0016		0.0006	0.0004	0.0027	2.6261	**0.009**
Smoking, %	13	−0.0001		0.0016	−0.0032	0.0031	−0.0357	0.972
Days at hospitalization (days)	11	0.0055		0.0060	−0.0062	0.0172	0.9275	0.354
ICU admission, %	9	0.0124		0.1495	−0.2807	0.3055	0.0828	0.934
Anxiety, %	17	0.4021		0.2916	−0.1695	0.9736	1.3788	0.168
Depression, %	17	−0.2669		0.2554	−0.7674	0.2337	−1.0449	0.296
Quality score	24	−0.0873		0.0357	−0.1572	−0.0174	−2.4469	**0.014**

CI, confidence interval; ICU, Intensive Care Unit; PSQI, Pittsburgh Sleep Quality Index. Bold values: *p* < 0.05.

### 3.5 Publication bias and sensitivity analyses

As shown in Supplementary Figures S2A,B, no publication bias was found in funnel plots. The Egger tests of the 36 studies (*t* = 1.41, *p* = 0.168) and the 12 comparative studies (*t* = 0.77, *p* = 0.457) also did not find any publication bias. Moreover, Supplementary Figures S4, S5 display the sensitivity analyses for the prevalence of poor sleep quality in the 36 studies and also the odds ratio in the 12 comparative studies, respectively. No outlying studies were found that could significantly alter the primary results.

## 4 Discussion

This is the first meta-analysis of the pooled prevalence of poor sleep quality in COVID-19 patients. The results showed that 65.0% (95%CI: 59.56–70.44%) of COVID-19 patients had poor sleep quality, which was around two times higher than that in healthy controls (OR = 1.73, 95%CI: 1.30–2.30). Globally, there were more than 757 million COVID-19 confirmed cases (59), which means that approximately 492 million COVID-19 patients suffered from poor sleep quality based on the prevalence rate found in this study. The COVID-19 pandemic had a profound impact on sleep quality in the population especially COVID-19 patients (11). A meta-analysis found that sleep problems were common and strongly associated with higher levels of psychological distress among health professionals, the general population, and COVID-19 patients during the COVID-19 pandemic (60). Another meta-analysis showed that sleep disturbances including poor sleep quality were higher among COVID-19 patients during lockdown periods compared with periods without lockdowns, and COVID-19 patients were the most affected subgroup (7). The high rate of sleep disturbances including poor sleep quality in COVID-19 patients may be related to several factors. First, sleep problems, including poor sleep quality, are among the main symptoms of COVID-19 infection (11). During the COVID-19 pandemic, the risk of mental health problems such as depression and anxiety increased, which had a negative impact on sleep quality of COVID-19 patients (27, 61). Second, those infected with COVID-19 are more likely to experience physical health problems (1, 2), such as fever, headache, dyspnea, myalgia, throat pain, cough, gastrointestinal disturbance, and other COVID-19 related symptoms (5, 6), which can affect immunological response and compromise sleep quality by altering related circadian rhythms (62). Moreover, many COVID-19 patients may suffer from impaired lung function and various sleep-related problems, such as obstructive sleep apnea, which affected sleep quality (63, 64). Third, usually COVID-19 patients are placed in long-term isolation treatment and tend to overuse their electronic devices during isolation causing chronic retinal irritation, which may lead to disturbance in sleep and circadian rhythm, and eventually poor sleep quality (65, 66). In addition, the strict lockdown measures can influence normal rhythms of the sleep–wake cycle among COVID-19 patients by reducing social activities and supports, increasing time spent in bed and delaying bedtimes (67, 68). As a result, depression, anxiety and insomnia are more likely to occur, thereby lowering their sleep quality (69, 70).

Subgroup analyses revealed that the prevalence of poor sleep quality in COVID-19 patients was higher in studies using PSQI cut-off value of 6 (69.5, 95% CI: 62.5–76.2%) compared to those using other PSQI cut-off values (e.g., cut-off of 8: 43.1, 95% CI: 32.2–54.1%), which is unsurprising given that a more stringent criterion for poor sleep quality would be associated with a lower prevalence rate. Poor sleep quality in COVID-19 patients in lower income countries (64.9, 95% CI: 62.6–67.2%) was more common than in higher income countries (e.g., upper middle income: 60.1, 95% CI: 58.1–62.2%; high income: 55.8, 95% CI: 55.4–57.2%), which could be explained by the fact that COVID-19 patients in low-income countries have less access to adequate and high-quality medical services compared to high-income countries (71). COVID-19 patients in high-income countries with advanced health system and greater resources tend to receive better care (72, 73) and have a lower mortality rate, which may also result in a lower risk of mental health problems such as anxiety, depression and insomnia among COVID-19 patients (72, 74, 75). In addition, both subgroup and meta-regression analyses revealed that women had a higher risk of poor sleep quality than men. This is consistent with previous findings that women are more likely to experience sleep problems than men such as poorer sleep quality, longer sleep latency periods, and more frequent use of sedative and hypnotic drugs (76). In addition, some studies found that women had more severe sleep difficulties than men during the COVID-19 pandemic due to shorter sleep duration and longer sleep latency (77, 78). This might be related to the finding that women are more susceptible than men to stress-related conditions (79, 80). A study conducted in Mexico found that the COVID-19 pandemic had different influences on women and men; for example, females reported more severe psychological distress, stress, and poorer sleep quality than males, probably related to the gender differences in response to stress, sensitivity to life events, and domestic and childcare responsibilities (81, 82).

Meta-regression analysis found more common poor sleep quality in older COVID-19 patients, which is consistent with previous research (83). There could be several reasons for this finding, first, older COVID-19 patients may have less outdoor activities and physical exercise, and spend more time in bed, leading to an increased risk of physical and psychological problems (84–86). Second, severe infection and morbidity rate are more common in older COVID-19 patients (87). Third, the likelihood of poor sleep quality and reduced sleep duration also increase with age. Meta-regression analysis also found that married COVID-19 patients had a higher risk of poor sleep quality. Compared to their unmarried counterparts, married COVID-19 patients may experience greater psychological pressure due to the fear of transmitting the virus to their family members, particularly children. Finally, higher quality studies were associated with a lower prevalence of poor sleep quality in COVID-19 patients. Stricter study design and more reliable measures were usually adopted in higher quality studies, which could reduce the likelihood of false positive rate and result in a lower prevalence of poor sleep quality.

The strengths of this meta-analysis included the large number of studies across different countries, inclusion of both epidemiological and comparative studies, and the use of sophisticated analyses such as subgroup, meta-regression, and sensitivity analyses. Additionally, all studies had used the PSQI to measure sleep quality, which would reduce the heterogeneity caused by different tools. However, several limitations of this study should be considered. First, due to the limited data, some factors related to sleep quality of COVID-19 patients could not be examined such as patients’ physical comorbidities, illness severity and treatment of COVID-19 patients. Second, due to differences in demographic data, COVID-19 related variables, sampling method and scale cutoff values between studies, heterogeneity could not be avoided in meta-analyses of epidemiological studies (88–91), even though subgroup analyses and meta-regression analyses were performed (92, 93). Finally, only published articles in English were included in this meta-analysis, which may bias the results of this study to an uncertain extent.

In conclusion, poor sleep quality was found to be very common in COVID-19 patients, especially in low-income countries, females, older adults and married patients. Considering the negative effects of poor sleep quality on daily life, sleep quality of COVID-19 patients should be assessed regularly, and effective treatments should be provided.

## Data availability statement

The original contributions presented in the study are included in the article/Supplementary material, further inquiries can be directed to the corresponding authors.

## Author contributions

ZG: Data curation, Formal Analysis, Funding acquisition, Investigation, Methodology, Resources, Software, Writing – original draft. Y-YW: Data curation, Writing – review & editing. J-XL: Data curation, Writing – review & editing. X-HL: Data curation, Writing – review & editing. ZS: Data curation, Writing – review & editing. TC: Data curation, Writing – review & editing. K-IL: Data curation, Writing – review & editing. GU: Data curation, Writing – review & editing. CN: Data curation, Writing – review & editing. QZ: Writing – review & editing, Methodology. Y-TX: Data curation, Formal Analysis, Investigation, Methodology, Project administration, Software, Supervision, Writing – review & editing.

## Footnotes

^1^ https://www.r-project.org

^2^https://www.worldbank.org
