# Supplementary material for: Prevalence of poor sleep quality in COVID-19 patients: a systematic review and meta-analysis
Source: Front Psychiatry. 2024 Jan 8;14:1272812. doi: 10.3389/fpsyt.2023.1272812 (PMC10800583; doi:10.3389/fpsyt.2023.1272812)
Supplement: Supplementary file 1 [file Data_Sheet_1.doc]

**Prevalence of poor sleep quality in COVID-19 patients: a systematic review and meta-analysis**

**Running head:** **Poor sleep quality in COVID-19 patients**

**Supplementary materials**

**Table S1.** Quality assessment of included studies

**Table S2.** Full-text articles excluded (n = 256)

**Figure S1.** PRISMA flowchart

**Table S3.** PRISMA 2020 Checklist

**Figure S2.** Funnel plot of publication bias for the included studies on prevalence of poor sleep quality A, Funnel plot of publication bias for 36 studies on prevalence of poor sleep quality (Egger’s test t = 1.41, p = 0.168). B, Funnel plot of publication bias for 12 studies on prevalence of poor sleep quality between COVID-19 patients and healthy controls (Egger’s test t = 0.77, p = 0.457).

**Figure S3.** Forest plot of PSQI total score in COVID-19 patients and healthy controls

**Figure S4.** Sensitivity analysis of pooled prevalence of poor sleep quality in COVID-19 patients

**Figure S5.** Sensitivity analysis of Odds Ratio for poor sleep quality between COVID-19 patients and healthy controls

**Table S1.** Quality assessment of included studies

| **Eight-Item assessment instrument for epidemiological studies** | | | | | | | | | |
| --- | --- | --- | --- | --- | --- | --- | --- | --- | --- |
| First author, Publication year | 1. Target population is clearly defined? | 2. Probability sampling OR entire population surveyed? | 3. Is the response rate ≥80%? | 4. Are non-responders clearly described? | 5. Is the sample representative of the target population? | 6. Were data collection methods standardized? | 7. Were validated criteria used to diagnose poor sleep quality? | 8. Are the prevalence estimates given with confidence intervals and detailed by subgroups (if applicable)? | Total score# |
| Ahmed et al., 2021(1) | 1 | 0 | 0 | 0 | 1 | 1 | 1 | 0 | 4 |
| Akinci et al., 2021(2) | 1 | 0 | 1 | 0 | 1 | 1 | 1 | 1 | 6 |
| Akova et al., 2022(3) | 1 | 0 | 0 | 0 | 1 | 1 | 1 | 1 | 5 |
| Awan et al., 2022(4) | 1 | 0 | 0 | 0 | 1 | 1 | 1 | 0 | 4 |
| Benitez et al., 2022(5) | 1 | 0 | 0 | 0 | 1 | 1 | 1 | 0 | 4 |
| Bungenberg et al., 2022(6) | 1 | 0 | 0 | 0 | 1 | 1 | 1 | 0 | 4 |
| Cacciatore et al., 2022(7) | 1 | 0 | 0 | 0 | 1 | 1 | 1 | 1 | 5 |
| Chhajer et al., 2022(8) | 1 | 0 | 0 | 0 | 1 | 1 | 1 | 0 | 4 |
| Choudhry et al., 2021(9) | 1 | 0 | 1 | 1 | 1 | 1 | 1 | 0 | 6 |
| Dai et al., 2020(10) | 1 | 0 | 0 | 0 | 1 | 1 | 1 | 1 | 5 |
| Delgado-Alonso et al., 2022(11) | 1 | 0 | 1 | 0 | 1 | 1 | 1 | 0 | 5 |
| Fernandez et al., 2022(12) | 1 | 0 | 0 | 0 | 1 | 1 | 1 | 0 | 4 |
| Fernandez et al., 2022(13) | 1 | 1 | 1 | 1 | 1 | 1 | 1 | 0 | 7 |
| Gundogdu et al., 2022(14) | 1 | 0 | 0 | 0 | 1 | 1 | 1 | 1 | 5 |
| Gunes et al., 2022(15) | 1 | 0 | 0 | 0 | 1 | 1 | 1 | 0 | 4 |
| Hartung et al., 2022(16) | 1 | 1 | 0 | 1 | 1 | 1 | 1 | 0 | 6 |
| Henriquez et al., 2022(17) | 1 | 0 | 0 | 0 | 1 | 1 | 1 | 0 | 4 |
| Li, X. et al., 2021(18) | 1 | 0 | 0 | 1 | 1 | 1 | 1 | 1 | 6 |
| Li, Z. et al., 2022(19) | 1 | 0 | 1 | 0 | 1 | 1 | 1 | 1 | 6 |
| Nowakowski et al., 2022(20) | 1 | 0 | 0 | 0 | 1 | 1 | 1 | 0 | 4 |
| Rousseau et al., 2021(21) | 1 | 0 | 0 | 1 | 1 | 1 | 1 | 0 | 5 |
| Tanriverdi et al., 2022(22) | 1 | 0 | 0 | 0 | 1 | 1 | 1 | 0 | 4 |
| Yadav et al., 2021(23) | 1 | 0 | 0 | 0 | 1 | 1 | 1 | 1 | 5 |
| Zhang, J et al., 2020(24) | 1 | 0 | 0 | 0 | 1 | 1 | 1 | 1 | 5 |
| **Newcastle-Ottawa quality assessment scale** | | | | | | | | | |
| First author, Publication year | 1.Is the  case definition adequate | 2.Representativeness of the cases | 3.Selection of Controls | 4.Definition of Controls | 5.Comparability | 6.Ascertainment of exposure | 7.Same method of ascertainment for cases and controls | 8.Non-Response rate | Total score* |
| Abbas et al., 2021(25) | 1 | 1 | 0 | 0 | 1 | 1 | 1 | 0 | 5 |
| Abdelghani et al., 2022(26) | 1 | 1 | 0 | 1 | 0 | 1 | 1 | 1 | 6 |
| Al-Ameri et al., 2022(27) | 1 | 1 | 0 | 1 | 1 | 1 | 1 | 0 | 6 |
| Al-Otaibi et al., 2022(28) | 1 | 1 | 0 | 0 | 0 | 1 | 1 | 0 | 4 |
| Alshumrani et al., 2022(29) | 1 | 1 | 0 | 0 | 0 | 1 | 1 | 0 | 4 |
| Amra et al., 2021(30) | 1 | 1 | 0 | 0 | 1 | 1 | 1 | 0 | 5 |
| Del Brutto et al., 2021(31) | 1 | 1 | 1 | 0 | 0 | 1 | 1 | 1 | 6 |
| ElHafeez et al., 2022(32) | 1 | 1 | 1 | 0 | 1 | 1 | 1 | 0 | 6 |
| Karaogullarindan et al., 2021(33) | 1 | 1 | 0 | 0 | 0 | 1 | 1 | 0 | 4 |
| Lin et al., 2022(34) | 1 | 1 | 1 | 0 | 1 | 1 | 1 | 0 | 6 |
| Malik et al., 2022(35) | 1 | 1 | 1 | 0 | 0 | 1 | 1 | 0 | 5 |
| Sljivo et al., 2022(36) | 1 | 1 | 1 | 0 | 0 | 1 | 1 | 0 | 5 |

Notes: Total score*, score of Newcastle-Ottawa Scale (NOS); Total score#, score of standardized instruments for epidemiological single arm studies

**Table S2.** Full-text articles excluded (n = 256)

| Excluded reason | Studies |
| --- | --- |
| No prevalence of poor sleep quality (n = 134) | (37-170) |
| No COVID-19 patients (n = 95) | (171-265) |
| Duplicate database (n = 8) | (266-273) |
| Commentaries/conference abstract/dissertations (n = 10) | (274-283) |
| Baseline data were unavailable for the cohort study (n = 9) | (284-292) |

**Table S3 PRISMA 2020 Checklist**

| **Section and Topic** | **Item #** | **Checklist item** | **Location where item is reported** |
| --- | --- | --- | --- |
| **TITLE** | | |  |
| Title | 1 | Identify the report as a systematic review. | Page |
| **ABSTRACT** | | |  |
| Abstract | 2 | See the PRISMA 2020 for Abstracts checklist. | Page |
| **INTRODUCTION** | | |  |
| Rationale | 3 | Describe the rationale for the review in the context of existing knowledge. | Page |
| Objectives | 4 | Provide an explicit statement of the objective(s) or question(s) the review addresses. | Page |
| **METHODS** | | |  |
| Eligibility criteria | 5 | Specify the inclusion and exclusion criteria for the review and how studies were grouped for the syntheses. | Page |
| Information sources | 6 | Specify all databases, registers, websites, organisations, reference lists and other sources searched or consulted to identify studies. Specify the date when each source was last searched or consulted. | Page |
| Search strategy | 7 | Present the full search strategies for all databases, registers and websites, including any filters and limits used. | Page |
| Selection process | 8 | Specify the methods used to decide whether a study met the inclusion criteria of the review, including how many reviewers screened each record and each report retrieved, whether they worked independently, and if applicable, details of automation tools used in the process. | Page |
| Data collection process | 9 | Specify the methods used to collect data from reports, including how many reviewers collected data from each report, whether they worked independently, any processes for obtaining or confirming data from study investigators, and if applicable, details of automation tools used in the process. | Page |
| Data items | 10a | List and define all outcomes for which data were sought. Specify whether all results that were compatible with each outcome domain in each study were sought (e.g. for all measures, time points, analyses), and if not, the methods used to decide which results to collect. | Page |
|  | 10b | List and define all other variables for which data were sought (e.g. participant and intervention characteristics, funding sources). Describe any assumptions made about any missing or unclear information. | Page |
| Study risk of bias assessment | 11 | Specify the methods used to assess risk of bias in the included studies, including details of the tool(s) used, how many reviewers assessed each study and whether they worked independently, and if applicable, details of automation tools used in the process. | Page |
| Effect measures | 12 | Specify for each outcome the effect measure(s) (e.g. risk ratio, mean difference) used in the synthesis or presentation of results. | Page |
| Synthesis methods | 13a | Describe the processes used to decide which studies were eligible for each synthesis (e.g. tabulating the study intervention characteristics and comparing against the planned groups for each synthesis (item #5)). | Page |
|  | 13b | Describe any methods required to prepare the data for presentation or synthesis, such as handling of missing summary statistics, or data conversions. | Page |
|  | 13c | Describe any methods used to tabulate or visually display results of individual studies and syntheses. | Page |
|  | 13d | Describe any methods used to synthesize results and provide a rationale for the choice(s). If meta-analysis was performed, describe the model(s), method(s) to identify the presence and extent of statistical heterogeneity, and software package(s) used. | Page |
|  | 13e | Describe any methods used to explore possible causes of heterogeneity among study results (e.g. subgroup analysis, meta-regression). | Page |
|  | 13f | Describe any sensitivity analyses conducted to assess robustness of the synthesized results. | Page |
| Reporting bias assessment | 14 | Describe any methods used to assess risk of bias due to missing results in a synthesis (arising from reporting biases). | Page |
| Certainty assessment | 15 | Describe any methods used to assess certainty (or confidence) in the body of evidence for an outcome. | Page |
| **RESULTS** | | |  |
| Study selection | 16a | Describe the results of the search and selection process, from the number of records identified in the search to the number of studies included in the review, ideally using a flow diagram. | Page , figure S1 |
|  | 16b | Cite studies that might appear to meet the inclusion criteria, but which were excluded, and explain why they were excluded. | Table S2 |
| Study characteristics | 17 | Cite each included study and present its characteristics. | Table 1 |
| Risk of bias in studies | 18 | Present assessments of risk of bias for each included study. | Figures S2a and S2b |
| Results of individual studies | 19 | For all outcomes, present, for each study: (a) summary statistics for each group (where appropriate) and (b) an effect estimate and its precision (e.g. confidence/credible interval), ideally using structured tables or plots. | Table 1 |
| Results of syntheses | 20a | For each synthesis, briefly summarise the characteristics and risk of bias among contributing studies. | Table 1, figure1 and Figures S2a and S2b |
|  | 20b | Present results of all statistical syntheses conducted. If meta-analysis was done, present for each the summary estimate and its precision (e.g. confidence/credible interval) and measures of statistical heterogeneity. If comparing groups, describe the direction of the effect. | Pages |
|  | 20c | Present results of all investigations of possible causes of heterogeneity among study results. | Page |
|  | 20d | Present results of all sensitivity analyses conducted to assess the robustness of the synthesized results. | Page , and Figures S2a and S2b |
| Reporting biases | 21 | Present assessments of risk of bias due to missing results (arising from reporting biases) for each synthesis assessed. | Page |
| Certainty of evidence | 22 | Present assessments of certainty (or confidence) in the body of evidence for each outcome assessed. | Pages |
| **DISCUSSION** | | |  |
| Discussion | 23a | Provide a general interpretation of the results in the context of other evidence. | Page |
|  | 23b | Discuss any limitations of the evidence included in the review. | Page |
|  | 23c | Discuss any limitations of the review processes used. | Page |
|  | 23d | Discuss implications of the results for practice, policy, and future research. | Pages |
| **OTHER INFORMATION** | | |  |
| Registration and protocol | 24a | Provide registration information for the review, including register name and registration number, or state that the review was not registered. | Page |
|  | 24b | Indicate where the review protocol can be accessed, or state that a protocol was not prepared. | Page |
|  | 24c | Describe and explain any amendments to information provided at registration or in the protocol. | Page |
| Support | 25 | Describe sources of financial or non-financial support for the review, and the role of the funders or sponsors in the review. | Page |
| Competing interests | 26 | Declare any competing interests of review authors. | Page |
| Availability of data, code and other materials | 27 | Report which of the following are publicly available and where they can be found: template data collection forms; data extracted from included studies; data used for all analyses; analytic code; any other materials used in the review. | Page |

Records identified through database searching (n=4,238):

-PubMed (n = 1,141);

-Web of Science (n = 1,487);

-EMBASE (n = 1,316);

-PsycINFO (n = 294)

Records removed before screening (n=2,289):

-Duplicate records removed

(n = 1,662)

-Records marked as ineligible by automation tools (n = 627)

Records after duplicates screened (n = 1,949)

Records excluded after reading title and abstract (n = 1,656)

Reports sought for retrieval

(n = 305)

Full-text articles assessed for eligibility (n = 293)

Full-text articles excluded (n = 256)

-No prevalence of poor sleep quality (n = 134)

-No COVID-19 patients (n = 95)

-Duplicate database (n = 8)

-Commentaries/conference abstract/dissertations (n = 10)

-Baseline data were unavailable for the cohort study (n = 9)

**Identification of new studies via databases and registers**

**Identification**

**Screening**

**Included**

Studies included in review

(n = 36)

Comparative studies (n = 11)

Studies included in previous version of review (n = 0)

Reports of studies included in previous version of review (n = 0)

**Previous studies**

Reports not retrieved (n = 12)

**Figure S1.** PRISMA flowchart

**
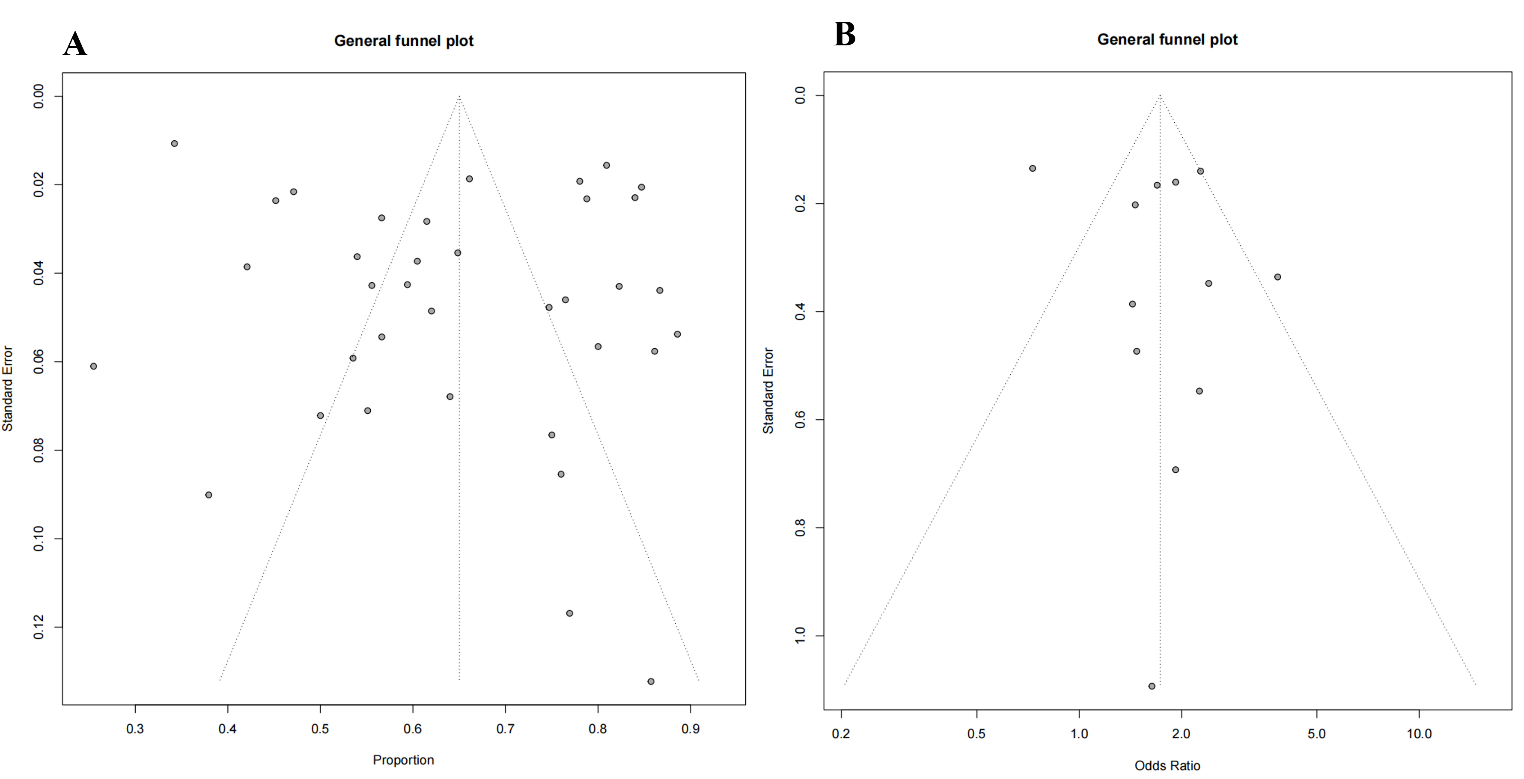
**

**Figure S2.** Funnel plot of publication bias for the included studies on prevalence of poor sleep quality. A, Funnel plot of publication bias for 36 studies on prevalence of poor sleep quality (Egger’s test t = 1.41, p = 0.168). B, Funnel plot of publication bias for 12 studies on prevalence of poor sleep quality between COVID-19 patients and healthy controls (Egger’s test t = 0.77, p = 0.457).

**
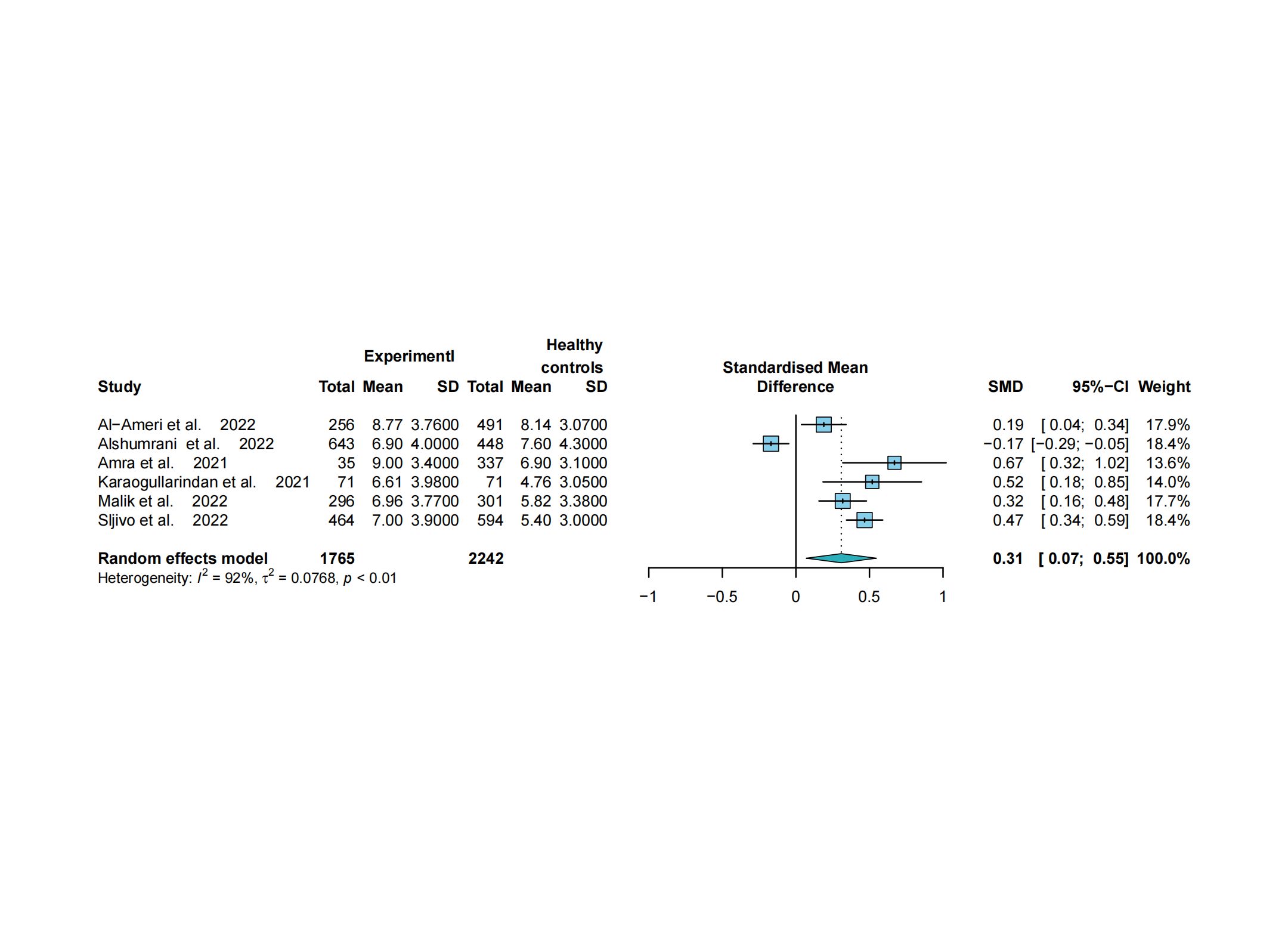
**

**Figure S3.** Forest plot of PSQI total score in COVID-19 patients and healthy controls

**
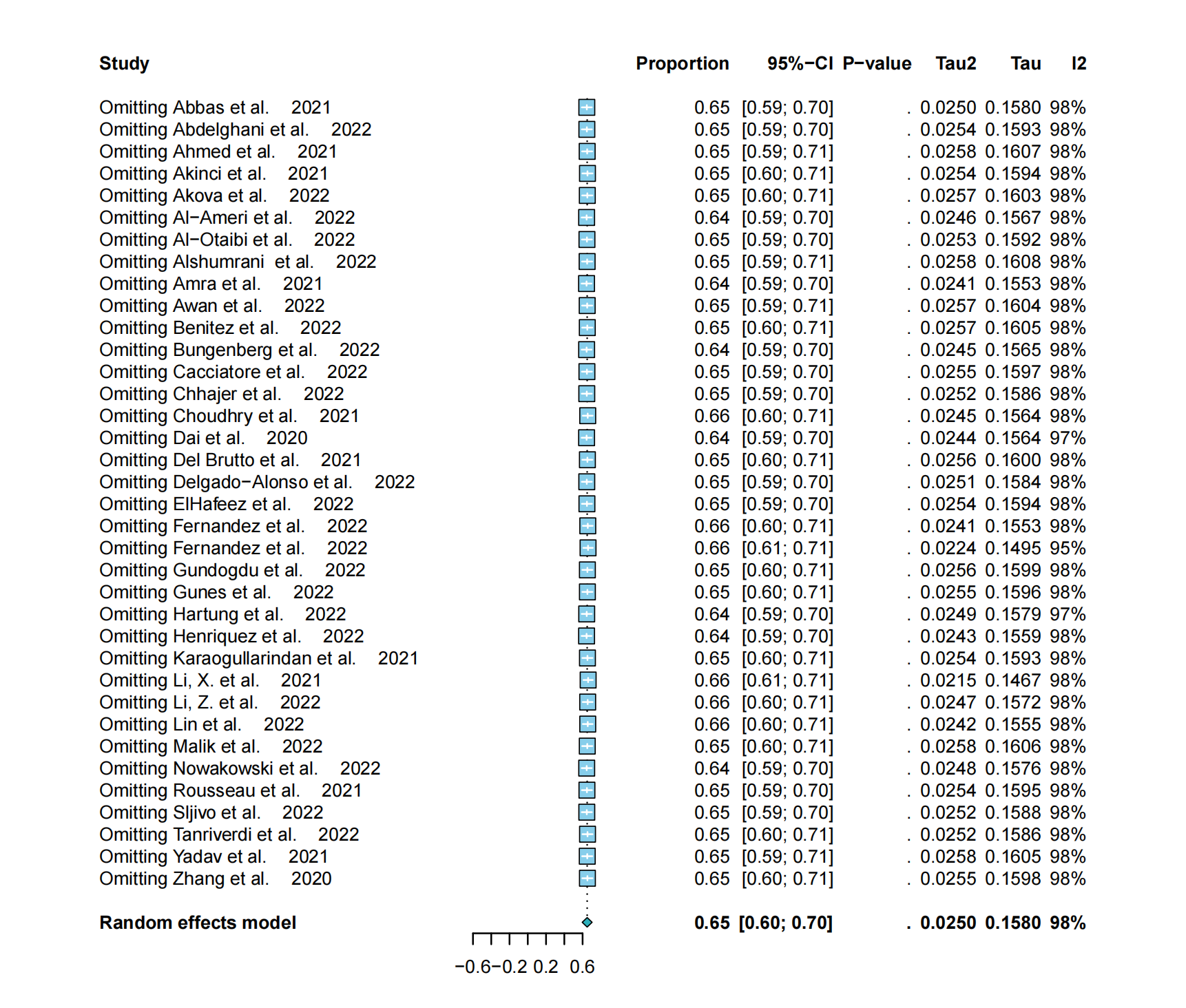
**

**Figure S4.** Sensitivity analysis of pooled prevalence of poor sleep quality in COVID-19 patients

**
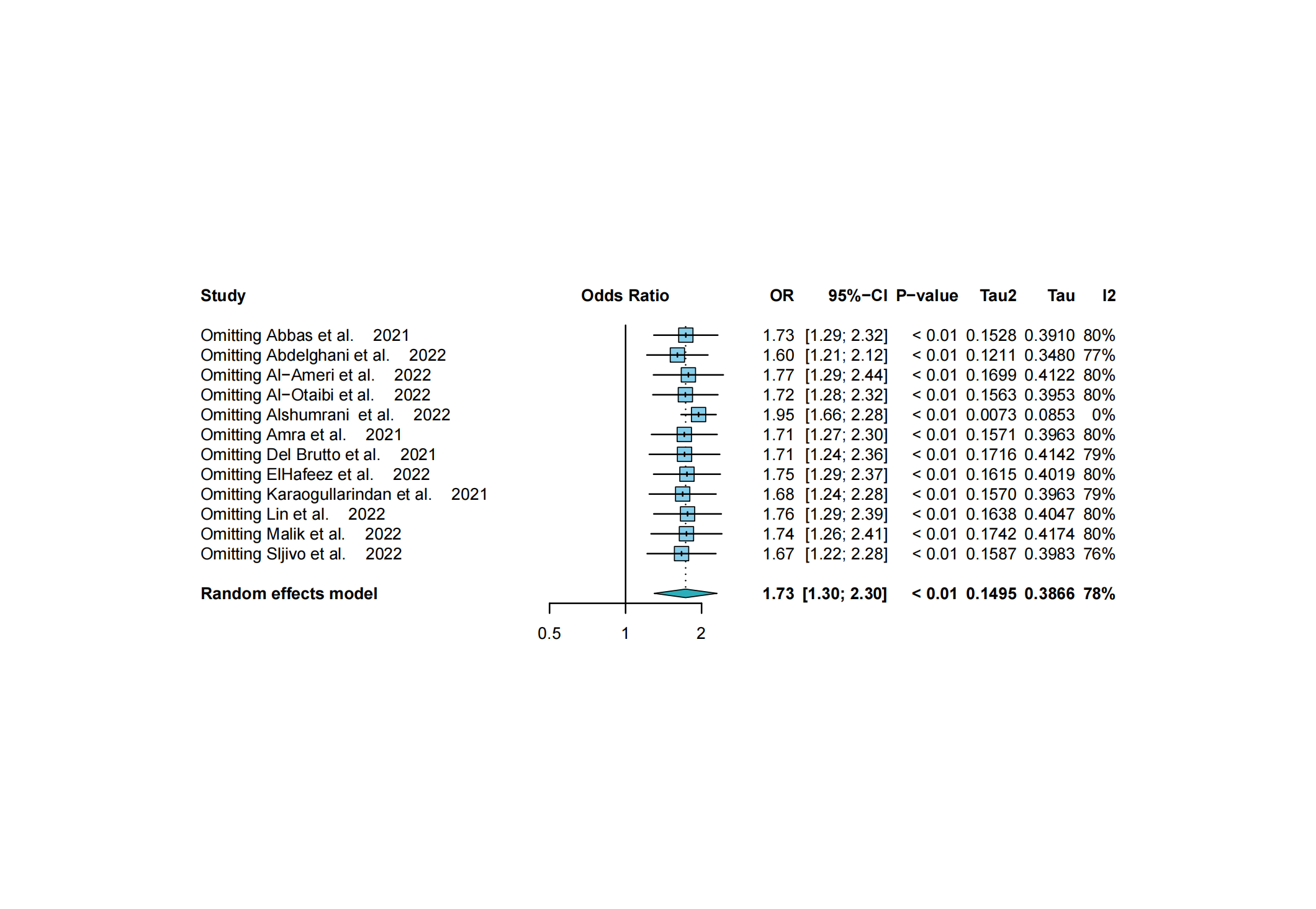
**

**Figure S5.** Sensitivity analysis of Odds Ratio for poor sleep quality between COVID-19 patients and healthy controls

**References**

1. Ahmed GK, Khedr EM, Hamad DA, Meshref TS, Hashem MM, Aly MM. Long term impact of Covid-19 infection on sleep and mental health: A cross-sectional study. Psychiatry Research. 2021;305:114243.

2. Akinci T, Melek Basar H. Relationship between sleep quality and the psychological status of patients hospitalised with COVID-19. Sleep Medicine. 2021;80:167-70.

3. Akova I, Gedikli MA. Fatigue and Sleep Quality Levels of Post-COVID-19 Healthcare Workers and Affecting Factors. International Journal of Academic Medicine and Pharmacy. 2022;4(1):65-9.

4. Awan I, Balouch AH, Juseja AK, Lakho MA, Shaikh A. Long Term Impact of COVID-19 Infection on Sleep and Mental Health. Pakistan Journal of Medical and Health Sciences. 2022;16(4):102-4.

5. Benitez ID, Moncusi-Moix A, Vaca R, Gort-Paniello C, Minguez O, Santisteve S, et al. Sleep and Circadian Health of Critical COVID-19 Survivors 3 Months After Hospital Discharge. Critical care medicine. 2022;50(6):945-54.

6. Bungenberg J, Humkamp K, Hohenfeld C, Rust MI, Ermis U, Dreher M, et al. Long COVID-19: Objectifying most self-reported neurological symptoms. Annals of Clinical and Translational Neurology. 2022;9(2):141-54.

7. Cacciatore M, Raggi A, Pilotto A, Cristillo V, Guastafierro E, Toppo C, et al. Neurological and Mental Health Symptoms Associated with Post-COVID-19 Disability in a Sample of Patients Discharged from a COVID-19 Ward: A Secondary Analysis. International Journal of Environmental Research and Public Health. 2022;19(7):4242.

8. Chhajer R, Shukla SD. Sleep Quality of Covid-19 Recovered Patients in India. Neuroscience Insights. 2022;17:9.

9. Choudhry AA, Shahzeen F, Choudhry SA, Batool N, Murtaza F, Dilip A, et al. Impact of COVID-19 Infection on Quality of Sleep. Cureus. 2021;13(9):e18182.

10. Dai LL, Wang X, Jiang TC, Li PF, Wang Y, Wu SJ, et al. Anxiety and depressive symptoms among COVID-19 patients in Jianghan Fangcang Shelter Hospital in Wuhan, China. PLoS ONE. 2020;15(8 august):e0238416.

11. Delgado-Alonso C, Valles-Salgado M, Delgado-Alvarez A, Yus M, Gomez-Ruiz N, Jorquera M, et al. Cognitive dysfunction associated with COVID-19: A comprehensive neuropsychological study. Journal of Psychiatric Research. 2022;150:40-6.

12. Fernández-de-Las-Peñas C, Cancela-Cilleruelo I, Moro-López-Menchero P, Rodríguez-Jiménez J, Gómez-Mayordomo V, Torres-Macho J, et al. Prevalence of Musculoskeletal Post-COVID Pain in Hospitalized COVID-19 Survivors Depending on Infection with the Historical, Alpha or Delta SARS-CoV-2 Variant. Biomedicines. 2022;10(8).

13. Fernandez-De-las-Penas C, de-La-Llave-Rincon AI, Ortega-Santiago R, Ambite-Quesada S, Gomez-Mayordomo V, Cuadrado ML, et al. Prevalence and risk factors of musculoskeletal pain symptoms as long-term post-COVID sequelae in hospitalized COVID-19 survivors: a multicenter study. Pain. 2022;163(9):E989-E96.

14. Gundogdu N, Tanriverdi M. Sleep Quality of Patients with COVID-19 after Hospital Discharge. Progress in Nutrition. 2022;24:e2022059.

15. Gunes A, Sensoy B. Sleepiness, Insomnia, and Sleep Quality of Hospitalized Patients with Coronavirus Disease-2019: Sleep Scale Evaluation. Journal of Turkish Sleep Medicine. 2022;9(1):11-5.

16. Hartung TJ, Neumann C, Bahmer T, Chaplinskaya-Sobol I, Endres M, Geritz J, et al. Fatigue and cognitive impairment after COVID-19: A prospective multicentre study. EClinicalMedicine. 2022;53:101651.

17. Henriquez-Beltran M, Labarca G, Cigarroa I, Enos D, Lastra J, Nova-Lamperti E, et al. Sleep health and the circadian rest-activity pattern four months after COVID-19. Jornal Brasileiro de Pneumologia. 2022;48(3):e20210398.

18. Li X, Cai Q, Jia Z, Liu L, Zhou Y, Zhang B, et al. The correlation between mental health status, sleep quality, and inflammatory markers, virus negative conversion time among patients confirmed with 2019-nCoV during the COVID-19 outbreak in China An observational study. Medicine (United States). 2021;100(27):e26520.

19. Li Z, He J, Wang Y, Bai M, Zhang Y, Chen H, et al. A cross-sectional study on the mental health of patients with COVID-19 1 year after discharge in Huanggang, China. European Archives of Psychiatry and Clinical Neuroscience. 2022.

20. Nowakowski S, Kokonda M, Sultana R, Duong BB, Nagy SE, Zaidan MF, et al. Association between Sleep Quality and Mental Health among Patients at a Post-COVID-19 Recovery Clinic. Brain Sciences. 2022;12(5):586.

21. Rousseau AF, Minguet P, Colson C, Kellens I, Chaabane S, Delanaye P, et al. Post-intensive care syndrome after a critical COVID-19: cohort study from a Belgian follow-up clinic. Annals of Intensive Care. 2021;11(1):118.

22. Tanriverdi A, Savci S, Kahraman BO, Ozpelit E. Extrapulmonary features of post-COVID-19 patients: muscle function, physical activity, mood, and sleep quality. Irish Journal of Medical Science. 2022;191(3):969-75.

23. Yadav R, Yadav P, Kumar SS, Kumar R. Assessment of Depression, Anxiety, and Sleep Disturbance in COVID-19 Patients at Tertiary Care Center of North India. Journal of Neurosciences in Rural Practice. 2021;12(2):316-22.

24. Zhang J, Xu D, Xie B, Zhang Y, Huang H, Liu H, et al. Poor-sleep is associated with slow recovery from lymphopenia and an increased need for ICU care in hospitalized patients with COVID-19: A retrospective cohort study. Brain, Behavior, and Immunity. 2020;88:50-8.

25. Abbas A, Al-Otaibi T, Gheith OA, Nagib AM, Farid MM, Walaa M. Sleep quality among healthcare workers during the covid-19 pandemic and its impact on medical errors: Kuwait experience. Turkish Thoracic Journal. 2021;22(2):142-8.

26. Abdelghani M, Alsadik M, Abdelmoaty A, Atwa S, Said A, Hassan M. Sleep disturbances following recovery from COVID-19: a comparative cross-sectional study in Egypt. Eastern Mediterranean Health Journal. 2022;28(1):14-22.

27. Al-Ameri LT, Hameed EK, Maroof BS. Sleep quality in COVID-19 recovered patients. Sleep Science. 2022;15(2):168-71.

28. Al-Otaibi T, Abbas A, Nagib AM, Gheith OA, Nair P, Farid MM, et al. COVID-somnia: anxiety, insomnia, and poor sleep among second-line healthcare workers during COVID-19 pandemic. EGYPTIAN JOURNAL OF BRONCHOLOGY. 2022;16(1).

29. Alshumrani R, Qanash S, Aldobyany A, Alhejaili F, Alqassas I, Shabrawishi M, et al. Sleep quality and mental health in coronavirus disease 2019 patients and general population during the pandemic. Annals of Thoracic Medicine. 2022;17(1):21-7.

30. Amra B, Salmasi M, Soltaninejad F, Sami R, Nickpour M, Mansourian M, et al. Healthcare workers' sleep and mood disturbances during COVID-19 outbreak in an Iranian referral center. Sleep and Breathing. 2021;25(4):2197-204.

31. Del Brutto OH, Mera RM, Costa AF, Recalde BY, Castillo PR. Sleep quality deterioration in middle-aged and older adults living in a rural Ecuadorian village severely struck by the SARS-CoV-2 pandemic. A population-based longitudinal prospective study. Sleep. 2021;44(8):6.

32. ElHafeez SA, Meira e Cruz M, Gouda S, Nofal M, Fayed A, Ghazy RM, et al. Sleep quality and anxiety among Egyptian population during covid-19 pandemic. Sleep Science. 2022;15(1):8-16.

33. Karaogullarindan A, Erkan SO, Tuhanioglu B, Kuran G, Gorgulu O. Sleep quality in patients over 65 years of age in the covid-19 pandemic. Turk Geriatri Dergisi. 2021;24(3):381-90.

34. Lin Y, Liu S, Li S, Zuo H, Zhang B. Relationships between the changes in sleep patterns and sleep quality among Chinese people during the 2019 coronavirus disease outbreak. Sleep Medicine. 2022;91:154-60.

35. Malik M, Atiq A, Tahir MJ, Akhtar FK, Malik MI, Hassan W, et al. Comparison of sleep quality among COVID-19 patients and non-COVID-19 population in Pakistan: A cross sectional study during the COVID-19 pandemic. Annals of Medicine and Surgery. 2022;78:103894.

36. Sljivo A, Juginovic A, Ivanovic K, Quraishi I, Mulac A, Kovacevic Z, et al. Sleep quality and patterns of young West Balkan adults during the third wave of COVID-19 pandemic: A cross-sectional study. BMJ Open. 2022;12(5):e060381.

37. Ahmad MS, Hicks SR, Watson R, Ahmed RA, Jones L, Vaselli M, et al. A patient satisfaction survey and educational package to improve the care of people hospitalised with COVID-19: a quality improvement project, Liverpool, UK. Wellcome open research. 2021;6:222.

38. Ahmadi MN, Huang BH, Inan-Eroglu E, Hamer M, Stamatakis E. Lifestyle risk factors and infectious disease mortality, including COVID-19, among middle aged and older adults: Evidence from a community-based cohort study in the United Kingdom. Brain, Behavior, and Immunity. 2021;96:18-27.

39. Akhlaghi A, Darabi A, Mahmoodi M, Movahed A, Kaboodkhani R, Mohammadi Z, et al. The Frequency and Clinical Assessment of COVID-19 in Patients With Chronic Rhinosinusitis. ENT-EAR NOSE & THROAT JOURNAL. 2021.

40. Aksan F, Nelson EA, Swedish KA. A COVID-19 patient with intense burning pain. JOURNAL OF NEUROVIROLOGY. 2020;26(5):800-1.

41. Al-Hashel JY, Ismail, II. Impact of coronavirus disease 2019 (COVID-19) pandemic on patients with migraine: a web-based survey study. JOURNAL OF HEADACHE AND PAIN. 2020;21(1).

42. Aloba O, Opakunle T. Fatigue and Sleep Quality Levels of Post-COVID-19 Healthcare Workers and Affecting Factors. International Medicine. 2021;3(5):145-51.

43. Alqahtani AS, Aldhahi MI, Alqahtani F, Altamimi M, Alshehri MM. Impact of the loss of smell on the sleep quality and fatigue level in COVID‑19 survivors. European archives of oto-rhino-laryngology : official journal of the European Federation of Oto-Rhino-Laryngological Societies (EUFOS) : affiliated with the German Society for Oto-Rhino-Laryngology - Head and Neck Surgery. 2022;279(9):4443-9.

44. Alshubaith IH, Alhajri S, Alhajri A, Alsultan RA, Azhar EI, Alhussaini BH, et al. The impact of COVID-19 on the sustainability of the environment, animal health and food security, and safety. ENVIRONMENTAL SCIENCE AND POLLUTION RESEARCH. 2022;29(47):70822-31.

45. An XD, Duan LY, Zhang YH, Jin D, Zhao SH, Zhou RR, et al. The three syndromes and six Chinese patent medicine study during the recovery phase of COVID-19. CHINESE MEDICINE. 2021;16(1).

46. Basishvili T, Oniani N, Sakhelashvili I, Eliozishvili M, Khizanashvili M, Arabidze M, et al. Insomnia, pre-sleep arousal, psychosocial factors and changes in sleep pattern during the second wave lockdown of the covid-19 pandemic in georgia. Brain Sciences. 2022;12(1):17.

47. Besnier F, Berube B, Malo J, Gagnon C, Gregoire CA, Juneau M, et al. Cardiopulmonary Rehabilitation in Long-COVID-19 Patients with Persistent Breathlessness and Fatigue: The COVID-Rehab Study. International Journal of Environmental Research and Public Health. 2022;19(7):4133.

48. Bolatturk OF, Soylu AC. Evaluation of cognitive, mental, and sleep patterns of post-acute COVID-19 patients and their correlation with thorax CT. Acta Neurologica Belgica. 2022.

49. Bologna C, Madonna P, Pone E. Efficacy of Prolonged-Release Melatonin 2 mg (PRM 2 mg) prescribed for insomnia in hospitalized patients for COVID-19: a retrospective observational study. Italian Journal of Medicine. 2022;16(SUPPL 1):17.

50. Bozan O, Atis SE, Cekmen B, Senturk M, Kalkan A. Healthcare workers' sleep quality after COVID-19 infection: A cross-sectional study. International Journal of Clinical Practice. 2021;75(11):e14772.

51. Case KR, Wang CP, Hosek MG, Lill SF, Howell AB, Taylor BS, et al. Health-related quality of life and social determinants of health following COVID-19 infection in a predominantly Latino population. JOURNAL OF PATIENT-REPORTED OUTCOMES. 2022;6(1).

52. Chakrabarti S. Mental Health in Hospitalised COVID 19 Patients in Quarantine During Second Wave in a South Indian Private Teaching Hospital. JOURNAL OF MULTIDISCIPLINARY HEALTHCARE. 2021;14:2777-89.

53. Cooper SM, Thomas A, Bamishigbin O. Black American Fathers Employed in Higher-Risk Contexts for Contracting COVID-19: Implications for Individual Wellbeing and Work-Family Spillover. American journal of men's health. 2021;15(2):15579883211005617.

54. Dal Santo F, Gonzalez-Blanco L, Rodriguez-Revuelta J, Marina Gonzalez PA, Paniagua G, Garcia-Alvarez L, et al. Early Impact of the COVID-19 Outbreak on Sleep in a Large Spanish Sample. Behavioral sleep medicine. 2022;20(3):100-15.

55. de Rus Jacquet A, Bogard S, Normandeau CP, Degroot C, Postuma RB, Dupre N, et al. Clinical perception and management of Parkinson's disease during the COVID-19 pandemic: A Canadian experience. Parkinsonism and Related Disorders. 2021;91:66-76.

56. Delgado-Alonso C, Valles-Salgado M, Delgado-Álvarez A, Gómez-Ruiz N, Yus M, Polidura C, et al. Examining Association of Personality Characteristics and Neuropsychiatric Symptoms in Post-COVID Syndrome. Brain sciences. 2022;12(2).

57. Diem L, Schwarzwald A, Friedli C, Hammer H, Gomes-Fregolente L, Warncke J, et al. Multidimensional phenotyping of the post-COVID-19 syndrome: A Swiss survey study. CNS NEUROSCIENCE & THERAPEUTICS.

58. Dini M, Poletti B, Tagini S, Reitano MR, Allocco E, Mazzocco K, et al. Resilience, Psychological Well-Being and Daily Functioning Following Hospitalization for Respiratory Distress Due to SARS-CoV-2 Infection. HEALTHCARE. 2021;9(9).

59. Dong C, Qiao Y, Shang C, Liao X, Yuan X, Cheng Q, et al. Non-contact screening system based for COVID-19 on XGBoost and logistic regression. Computers in Biology and Medicine. 2022;141:105003.

60. Donzella SM, Kohler LN, Crane TE, Jacobs ET, Ernst KC, Bell ML, et al. COVID-19 Infection, the COVID-19 Pandemic, and Changes in Sleep. Frontiers in public health. 2021;9:795320.

61. Drager LF, Pachito DV, Moreno CRC, Tavares AR, Conway SG, Assis M, et al. Insomnia episodes, new-onset pharmacological treatments, and other sleep disturbances during the COVID-19 pandemic: a nationwide cross-sectional study in Brazilian health care professionals. Journal of Clinical Sleep Medicine. 2022;18(2):373-82.

62. Duncan DT, Park SH, Chen YT, Mountcastle H, Pagkas-Bather J, Timmins L, et al. Sleep characteristics among black cisgender sexual minority men and black transgender women during the COVID-19 pandemic: The role of multi-level COVID-19-related stressors. Sleep health. 2022.

63. e Silva NSS, Barbosa REC, Leão LL, das Graças Pena G, de Pinho L, de Magalhães TA, et al. Working conditions, lifestyle and mental health of Brazilian public-school teachers during the COVID-19 pandemic. Psychiatriki. 2021;32(4):282-9.

64. El Sayed S, Gomaa S, Shokry D, Kabil A, Eissa A. Sleep in post-COVID-19 recovery period and its impact on different domains of quality of life. Egyptian Journal of Neurology, Psychiatry and Neurosurgery. 2021;57(1):172.

65. Elhadi M, Alsoufi A, Msherghi A, Alshareea E, Ashini A, Nagib T, et al. Psychological Health, Sleep Quality, Behavior, and Internet Use Among People During the COVID-19 Pandemic: A Cross-Sectional Study. Frontiers in Psychiatry. 2021;12:632496.

66. Fakili LF, Bayram N. Anxiety Levels, Sleep Quality and Follow-up of Obstructive Sleep Apnoea Patients During the COVID-19 Pandemic. Journal of Turkish Sleep Medicine. 2022;9(2):147-52.

67. Fernandez-de-las-Penas C, Fuensalida-Novo S, Ortega-Santiago R, Valera-Calero JA, Cescon C, Derboni M, et al. Pain Extent Is Not Associated with Sensory-Associated Symptoms, Cognitive or Psychological Variables in COVID-19 Survivors Suffering from Post-COVID Pain. Journal of Clinical Medicine. 2022;11(15):4633.

68. Fernandez-de-las-Penas C, Giordano R, Diaz-Gil G, Gil-Crujera A, Gomez-Sanchez SM, Ambite-Quesada S, et al. Are Pain Polymorphisms Associated with the Risk and Phenotype of Post-COVID Pain in Previously Hospitalized COVID-19 Survivors? Genes. 2022;13(8):1336.

69. Fernandez-de-las-Penas C, Giordano R, Diaz-Gil G, Gomez-Esquer F, Ambite-Quesada S, Palomar-Gallego MA, et al. Post-COVID Pain Is Not Associated with Inflammatory Polymorphisms in People Who Had Been Hospitalized by COVID-19. Journal of Clinical Medicine. 2022;11(19):5645.

70. Fernandez-De-las-penas C, Herrero-Montes M, Cancela-Cilleruelo I, Rodriguez-Jimenez J, Paras-Bravo P, Varol U, et al. Understanding Sensitization, Cognitive and Neuropathic Associated Mechanisms behind Post-COVID Pain: A Network Analysis. Diagnostics. 2022;12(7):1538.

71. Fernandez-de-las-Penas C, Martin-Guerrero JD, Florencio LL, Navarro-Pardo E, Rodriguez-Jimenez J, Torres-Macho J, et al. Clustering analysis reveals different profiles associating long-term post-COVID symptoms, COVID-19 symptoms at hospital admission and previous medical co-morbidities in previously hospitalized COVID-19 survivors. Infection. 2022.

72. Fernandez-de-las-Penas C, Paras-Bravo P, Ferrer-Pargada D, Cancela-Cilleruelo I, Rodriguez-Jimenez J, Nijs J, et al. Sensitization symptoms are associated with psychological and cognitive variables in COVID-19 survivors exhibiting post-COVID pain. Pain Practice. 2022.

73. Fidanci I, Aksoy H, Taci DY, Fidanci I, Baser DA, Cankurtaran M. Evaluation of the effect of the COVID-19 pandemic on sleep disorders and nutrition in children. INTERNATIONAL JOURNAL OF CLINICAL PRACTICE. 2021;75(7).

74. Fiore V, De Vito A, Fanelli C, Geremia N, Princic E, Nivoli A, et al. Mood Reactive Disorders among COVID-19 Inpatients: Experience from a Monocentric Cohort. Medical Principles and Practice. 2021;30(6):535-41.

75. Frontera JA, Yang D, Medicherla C, Baskharoun S, Bauman K, Bell L, et al. Trajectories of Neurologic Recovery 12 Months After Hospitalization for COVID-19 A Prospective Longitudinal Study. NEUROLOGY. 2022;99(1):E33-E45.

76. Fu L, Fang Y, Luo D, Wang B, Xiao X, Hu Y, et al. Pre-hospital, in-hospital and post-hospital factors associated with sleep quality among COVID-19 survivors 6 months after hospital discharge: Cross-sectional survey in five cities in China. BJPsych Open. 2021;7(6):e191.

77. Fu L, Wang B, Chan PSF, Luo D, Zheng W, Ju N, et al. Associations between COVID-19 related stigma and sleep quality among COVID-19 survivors six months after hospital discharge. Sleep Medicine. 2022;91:273-81.

78. Garg A, Goyal S, Comellas AP. Post-acute COVID-19 functional movement disorder. SAGE OPEN MEDICAL CASE REPORTS. 2021;9.

79. Gonzalez-Hijon J, Kahler AK, Frans EM, Valdimarsdottir UA, Sullivan PF, Fang F, et al. Unravelling the link between sleep and mental health during the COVID-19 pandemic. medRxiv. 2022.

80. Gramaglia C, Gambaro E, Bellan M, Balbo PE, Baricich A, Sainaghi PP, et al. Mid-term Psychiatric Outcomes of Patients Recovered From COVID-19 From an Italian Cohort of Hospitalized Patients. FRONTIERS IN PSYCHIATRY. 2021;12.

81. Grazzini M, Lulli LG, Mucci N, Paolini D, Baldassarre A, Gallinoro V, et al. Return to Work of Healthcare Workers after SARS-CoV-2 Infection: Determinants of Physical and Mental Health. International Journal of Environmental Research and Public Health. 2022;19(11):6811.

82. Gundogdu N, Demirguc A, Cicek C, Ergun N. Evaluation of the relationship between the disease severity and the level of physical activity in patients followed up with COVID-19 diagnosis. Saudi Medical Journal. 2022;43(6):579-86.

83. Gungor S, Tosun B, Unal N, Dusak I. Evaluation of dyspnea severity and sleep quality in patients with novel coronavirus. International Journal of Clinical Practice. 2021;75(10):e14631.

84. Haider S, Janowski AJ, Lesnak JB, Hayashi K, Dailey DL, Chimenti R, et al. A comparison of pain, fatigue, and function between post-COVID-19 condition, fibromyalgia, and chronic fatigue syndrome: a survey study. Pain. 2022.

85. Han J, Qian K, Song MS, Yang ZJ, Ren Z, Liu S, et al. An Early Study on Intelligent Analysis of Speech under COVID-19: Severity, Sleep Quality, Fatigue, and Anxiety. INTERSPEECH 20202020. p. 4946-50.

86. Harenwall S, Heywood-Everett S, Henderson R, Godsell S, Jordan S, Moore A, et al. Post-Covid-19 Syndrome: Improvements in Health-Related Quality of Life Following Psychology-Led Interdisciplinary Virtual Rehabilitation. JOURNAL OF PRIMARY CARE AND COMMUNITY HEALTH. 2021;12.

87. Hay JW, Gong CL, Jiao XY, Zawadzki NK, Zawadzki RS, Pickard AS, et al. A US Population Health Survey on the Impact of COVID-19 Using the EQ-5D-5L. JOURNAL OF GENERAL INTERNAL MEDICINE. 2021;36(5):1292-301.

88. Heyen JM, Weigl N, Müller M, Müller S, Eberle U, Manoliu A, et al. Multimodule Web-Based COVID-19 Anxiety and Stress Resilience Training (COAST): Single-Cohort Feasibility Study With First Responders. JMIR formative research. 2021;5(6):e28055.

89. Huancahuire-Vega S, Newball-Noriega EE, Rojas-Humpire R, Saintila J, Rodriguez-Vasquez M, Ruiz-Mamani PG, et al. Changes in Eating Habits and Lifestyles in a Peruvian Population during Social Isolation for the COVID-19 Pandemic. Journal of Nutrition and Metabolism. 2021;2021:4119620.

90. Huang CL, Huang LX, Wang YM, Li X, Ren LL, Gu XY, et al. 6-month consequences of COVID-19 in patients discharged from hospital: a cohort study. LANCET. 2021;397(10270):220-32.

91. Huang LQ, Xu XH, Zhang LJ, Zheng DW, Liu YT, Feng B, et al. Post-traumatic Stress Disorder Symptoms and Quality of Life of COVID-19 Survivors at 6-Month Follow-Up: A Cross-Sectional Observational Study. FRONTIERS IN PSYCHIATRY. 2022;12.

92. Huhn AS, Strain EC, Jardot J, Turner G, Bergeria CL, Nayak S, et al. Treatment Disruption and Childcare Responsibility as Risk Factors for Drug and Alcohol Use in Persons in Treatment for Substance Use Disorders During the COVID-19 Crisis. JOURNAL OF ADDICTION MEDICINE. 2022;16(1):E8-E15.

93. Jagadeesan T, R A, R K, Jain T, Allu AR, Selvi GT, et al. Effect of Bhramari Pranayama intervention on stress, anxiety, depression and sleep quality among COVID 19 patients in home isolation. Journal of Ayurveda and Integrative Medicine. 2022;13(3):100596.

94. Jiang Z, Zhu P, Wang L, Hu Y, Pang M, Ma S, et al. Psychological distress and sleep quality of COVID-19 patients in Wuhan, a lockdown city as the epicenter of COVID-19. Journal of Psychiatric Research. 2021;136:595-602.

95. Kantorski LP, Oliveira MM, Alves PF, Treichel CADS, Coimbra VCC, Goncalves BA, et al. Prevalence and factors associated with poor sleep quality among nursing professionals during the COVID-19 pandemic. Revista brasileira de enfermagem. 2022;75Suppl 1(Suppl 1):e20210517.

96. Kleszczynski K, Slominski AT, Steinbrink K, Reiter RJ. Clinical trials for use of melatonin to fight against COVID-19 are urgently needed. Nutrients. 2020;12(9):1-12.

97. Knight DRT, Munipalli B, Logvinov II, Halkar MG, Mitri G, Dabrh AMA, et al. Perception, Prevalence, and Prediction of Severe Infection and Post-acute Sequelae of COVID-19. American Journal of the Medical Sciences. 2022;363(4):295-304.

98. Kong G, Kong D, Shi L. Sleeplessness in COVID-19 pandemic: Lockdown and anxiety. Journal of Asian economics. 2022;80:101460.

99. Kow CS, Hasan SS. Do sleep quality and sleep duration before or after COVID-19 vaccination affect antibody response? Chronobiology international. 2021;38(7):941-3.

100. Kumar N, Goyal A, Hussain A, Saini L, Chawla O, Arora P, et al. SARS-CoV-2 infection is associated with increased odds of insomnia, RLS and dream enactment behavior. Indian Journal of Psychiatry. 2022;64(4):354-63.

101. Lemhofer C, Sturm C, Loudovici-Krug D, Best N, Gutenbrunner C. The impact of Post-COVID-Syndrome on functioning - results from a community survey in patients after mild and moderate SARS-CoV-2-infections in Germany. JOURNAL OF OCCUPATIONAL MEDICINE AND TOXICOLOGY. 2021;16(1).

102. Lindahl A, Aro M, Reijula J, Makela MJ, Ollgren J, Puolanne M, et al. Women report more symptoms and impaired quality of life: a survey of Finnish COVID-19 survivors. INFECTIOUS DISEASES. 2022;54(1):53-62.

103. Liu X, Lin H, Jiang H, Li R, Zhong N, Su H, et al. Clinical characteristics of hospitalised patients with schizophrenia who were suspected to have coronavirus disease (COVID-19) in Hubei Province, China. General psychiatry. 2020;33(2):e100222.

104. Magnavita N, Tripepi G, Di Prinzio RR. Symptoms in health care workers during the covid-19 epidemic. A cross-sectional survey. International Journal of Environmental Research and Public Health. 2020;17(14):1-15.

105. Mahmoudi H, Saffari M, Movahedi M, Sanaeinasab H, Rashidi-Jahan H, Pourgholami M, et al. A mediating role for mental health in associations between COVID-19-related self-stigma, PTSD, quality of life, and insomnia among patients recovered from COVID-19. BRAIN AND BEHAVIOR. 2021;11(5).

106. Marchese MR, Cefaro CA, Giorgia M, Ilaria P, Angelo C, Matteo T, et al. Oropharyngeal Dysphagia After Hospitalization for COVID-19 Disease: Our Screening Results. DYSPHAGIA. 2022;37(2):447-53.

107. Margalit I, Yelin D, Sagi M, Rahat MM, Sheena L, Mizrahi N, et al. Risk factors and multidimensional assessment of long COVID fatigue: a nested case-control study. Clinical infectious diseases : an official publication of the Infectious Diseases Society of America. 2022.

108. Mariniello A, Schiavolin S, Magnani F, Cristillo V, Piccinelli SC, Zoppi N, et al. Neurological involvement associated with COVID-19 disease: a study on psychosocial factors. Neurological Sciences. 2022;43(4):2187-93.

109. Marogna C, Montanari E, Contiero S, Lleshi K. Dreaming during COVID-19: The effects of a world trauma. Research in Psychotherapy: Psychopathology, Process and Outcome. 2021;24(2):188-99.

110. Martins S, Ferreira AR, Fernandes J, Vieira T, Fontes L, Coimbra I, et al. Depressive and Anxiety Symptoms in Severe COVID-19 Survivors: A Prospective Cohort Study. PSYCHIATRIC QUARTERLY. 2022;93(3):891-903.

111. Matta J, Wiernik E, Robineau O, Carrat F, Touvier M, Severi G, et al. Association of Self-reported COVID-19 Infection and SARS-CoV-2 Serology Test Results With Persistent Physical Symptoms Among French Adults During the COVID-19 Pandemic. JAMA INTERNAL MEDICINE. 2022;182(1):19-25.

112. Mazza MG, Palladini M, Poletti S, Benedetti F. Post-COVID-19 Depressive Symptoms: Epidemiology, Pathophysiology, and Pharmacological Treatment. CNS Drugs. 2022;36(7):681-702.

113. Mekhael M, Lim CH, El Hajjar AH, Noujaim C, Pottle C, Makan N, et al. Studying the Effect of Long COVID-19 Infection on Sleep Quality Using Wearable Health Devices: Observational Study. Journal of Medical Internet Research. 2022;24(7):e38000.

114. Meller FO, Schafer AA, Quadra MR, Demenech LM, Paludo SDS, da Silva PA, et al. Fear of Covid-19 and health-related outcomes: results from two Brazilian population-based studies. Psychiatry Research. 2022;313:114596.

115. Meng ZQ, Leng Q, Wang X, Xu CH, Xu J, Liu Y. Comparative analysis of anxiety/depression scores in COVID-19 patients with disease severity, sleep patterns, and certain laboratory test results. Archives of medical science : AMS. 2022;18(5):1262-70.

116. Neville TH, Hays RD, Tseng CH, Gonzalez CA, Chen L, Hong A, et al. Survival After Severe COVID-19: Long-Term Outcomes of Patients Admitted to an Intensive Care Unit. JOURNAL OF INTENSIVE CARE MEDICINE. 2022;37(8):1019-28.

117. Orru G, Bertelloni D, Diolaiuti F, Mucci F, Di Giuseppe M, Biella M, et al. A Study on the Persistence of Neurological, Psychological and Physiological Symptoms. HEALTHCARE. 2021;9(5).

118. Ozkeskin M, Ozden F, Karaman B, Ekmekci O, Yuceyar N. The comparison of fatigue, sleep quality, physical activity, quality of life, and psychological status in multiple sclerosis patients with or without COVID-19. Multiple Sclerosis and Related Disorders. 2021;55:103180.

119. Pappa S, Barmparessou Z, Athanasiou N, Sakka E, Eleftheriou K, Patrinos S, et al. Depression, Insomnia and Post-Traumatic Stress Disorder in COVID-19 Survivors: Role of Gender and Impact on Quality of Life. JOURNAL OF PERSONALIZED MEDICINE. 2022;12(3).

120. Partinen M, Holzinger B, Morin CM, Espie C, Chung F, Penzel T, et al. Sleep and daytime problems during the COVID-19 pandemic and effects of coronavirus infection, confinement and financial suffering: A multinational survey using a harmonised questionnaire. BMJ Open. 2021;11(12):e050672.

121. Paul E, Fancourt D. Health behaviours the month prior to COVID-19 infection and the development of self-reported long COVID and specific long COVID symptoms: a longitudinal analysis of 1581 UK adults. BMC public health. 2022;22(1):1716.

122. Paul E, Fancourt D. Health behaviours the month prior to COVID-19 infection and the development of self-reported long COVID and specific long COVID symptoms: A longitudinal analysis of 1,811 UK adults. medRxiv. 2022.

123. Peltz JS. Mediators of the association between COVID-19-related stressors and parents' psychological flexibility and inflexibility: The roles of perceived sleep quality and energy (vol 17, pg 168, 2020). JOURNAL OF CONTEXTUAL BEHAVIORAL SCIENCE. 2021;21:224-.

124. Peluso MJ, Kelly JD, Lu S, Goldberg SA, Davidson MC, Mathur S, et al. Persistence, Magnitude, and Patterns of Postacute Symptoms and Quality of Life Following Onset of SARS-CoV-2 Infection: Cohort Description and Approaches for Measurement. OPEN FORUM INFECTIOUS DISEASES. 2022;9(2).

125. Qian K, Schmitt M, Zheng H, Koike T, Han J, Liu J, et al. Computer Audition for Fighting the SARS-CoV-2 Corona Crisis-Introducing the Multitask Speech Corpus for COVID-19. IEEE internet of things journal. 2021;8(21):16035-46.

126. Radhakrishnan NS, Mufti M, Ortiz D, Maye ST, Melara J, Lim D, et al. Implementing Delirium Prevention in the Era of COVID-19. JOURNAL OF ALZHEIMERS DISEASE. 2021;79(1):31-6.

127. Ramirez-Del Real T, Martinez-Garcia M, Marquez MF, Lopez-Trejo L, Gutierrez-Esparza G, Hernandez-Lemus E. Individual Factors Associated With COVID-19 Infection: A Machine Learning Study. Frontiers in public health. 2022;10:912099.

128. Ramos-Echevarria PM, Soto-Soto DM, Torres-Reveron A, Appleyard CB, Akkawi T, Barros-Cartagena BD, et al. Impact of the early COVID-19 era on endometriosis patients: Symptoms, stress, and access to care. JOURNAL OF ENDOMETRIOSIS AND PELVIC PAIN DISORDERS. 2021;13(2):111-21.

129. Rass V, Ianosi BA, Zamarian L, Beer R, Sahanic S, Lindner A, et al. Factors associated with impaired quality of life three months after being diagnosed with COVID-19. QUALITY OF LIFE RESEARCH. 2022;31(5):1401-14.

130. Rowlands AV, Kloecker DE, Chudasama Y, Davies MJ, Dawkins NP, Edwardson CL, et al. Association of Timing and Balance of Physical Activity and Rest/Sleep With Risk of COVID-19: A UK Biobank Study. Mayo Clinic Proceedings. 2021;96(1):156-64.

131. Rozmiarek M, Leon-Guereno P, Tapia-Serrano MA, Thuany M, Gomes TN, Ploszaj K, et al. Motivation and Eco-Attitudes among Night Runners during the COVID-19 Pandemic. SUSTAINABILITY. 2022;14(3).

132. Rubega M, Ciringione L, Bertuccelli M, Paramento M, Sparacino G, Vianello A, et al. High-density EEG sleep correlates of cognitive and affective impairment at 12-month follow-up after COVID-19. CLINICAL NEUROPHYSIOLOGY. 2022;140:126-35.

133. Rupp K, Friel CP. Changes in Health Behaviors Associated With Weight Gain by Weight Classification During the COVID-19 Pandemic. American journal of health promotion : AJHP. 2022;36(1):21-8.

134. Rydwik E, Anmyr L, Regardt M, McAllister A, Zarenoe R, Akerman E, et al. ReCOV: recovery and rehabilitation during and after COVID-19-a study protocol of a longitudinal observational study on patients, next of kin and health care staff. BMC SPORTS SCIENCE MEDICINE AND REHABILITATION. 2021;13(1).

135. Sahin F, Karadag F, Kucukkarapinar M. A Cross-Sectional Study Investigating Mental Health Among Turkish Citizens During The COVID-19 Pandemic: The Importance of Perceived Social Support. PSYCHIATRY AND BEHAVIORAL SCIENCES. 2022;12(1):14-25.

136. Salaffi F, Giorgi V, Sirotti S, Bongiovanni S, Farah S, Bazzichi L, et al. The effect of novel coronavirus disease-2019 (COVID-19) on fibromyalgia syndrome. Clinical and Experimental Rheumatology. 2021;39(4):S72-S7.

137. Salehinejad MA, Azarkolah A, Ghanavati E, Nitsche MA. Circadian disturbances, sleep difficulties and the COVID-19 pandemic. Sleep Medicine. 2022;91:246-52.

138. Scarpelli S, Nadorff MR, Bjorvatn B, Chung F, Dauvilliers Y, Espie CA, et al. Nightmares in People with COVID-19: Did Coronavirus Infect Our Dreams? NATURE AND SCIENCE OF SLEEP. 2022;14:93-108.

139. Seessle J, Waterboer T, Hippchen T, Simon J, Kirchner M, Lim A, et al. Persistent Symptoms in Adult Patients 1 Year After Coronavirus Disease 2019 (COVID-19): A Prospective Cohort Study. CLINICAL INFECTIOUS DISEASES. 2022;74(7):1191-8.

140. Sfera A, Osorio C, del Campo C, Pereida S, Maurer S, Maldonado JC, et al. Endothelial Senescence and Chronic Fatigue Syndrome, a COVID-19 Based Hypothesis. FRONTIERS IN CELLULAR NEUROSCIENCE. 2021;15.

141. Shah R, Ali FM, Nixon SJ, Ingram JR, Salek SM, Finlay AY. Measuring the impact of COVID-19 on the quality of life of the survivors, partners and family members: a cross-sectional international online survey. BMJ OPEN. 2021;11(5).

142. Shanshal SA, Al-Qazaz HK. The impact of COVID-19 infection on the quality of life of healthcare workers. JOURNAL OF PHARMACEUTICAL HEALTH SERVICES RESEARCH. 2022;13(2):89-94.

143. Shi L, Zhang D, Martin E, Chen Z, Li H, Han X, et al. Racial Discrimination, Mental Health and Behavioral Health During the COVID-19 Pandemic: a National Survey in the United States. Journal of General Internal Medicine. 2022;37(10):2496-504.

144. Siste K, Hanafi E, Sen LT, Christian H, Adrian, Siswidiani LP, et al. The Impact of Physical Distancing and Associated Factors Towards Internet Addiction Among Adults in Indonesia During COVID-19 Pandemic: A Nationwide Web-Based Study. Frontiers in Psychiatry. 2020;11:580977.

145. Soriano EC, Perndorfer C, Otto AK, Fenech AL, Siegel SD, Dickson-Witmer D, et al. Psychosocial Impact of Cancer Care Disruptions in Women With Breast Cancer During the COVID-19 Pandemic. FRONTIERS IN PSYCHOLOGY. 2021;12.

146. Sun L, Sun Z, Wu L, Zhu Z, Zhang F, Shang Z, et al. Prevalence and risk factors for acute posttraumatic stress disorder during the COVID-19 outbreak. Journal of Affective Disorders. 2021;283:123-9.

147. Suresh H, Nagaraja MS. A study of clinical profile, sequelae of COVID, and satisfaction of inpatient care at a government COVID care hospital in Karnataka. JOURNAL OF FAMILY MEDICINE AND PRIMARY CARE. 2022;11(6):2672-7.

148. t Hoog S, Eskes AM, van Oers JAH, Boerrigter JL, Prins-Smulders M, Oomen M, et al. A Quality Improvement Project to Support Post-Intensive Care Unit Patients with COVID-19: Structured Telephone Support. INTERNATIONAL JOURNAL OF ENVIRONMENTAL RESEARCH AND PUBLIC HEALTH. 2022;19(15).

149. Tahir MJ, Malik NI, Ullah I, Khan HR, Perveen S, Ramalho R, et al. Internet addiction and sleep quality among medical students during the COVID-19 pandemic: A multinational cross-sectional survey. PLoS ONE. 2021;16(11 November):e0259594.

150. Tapan OO, Tapan U, Alasan F, Akgul AF, Genc S. Factors That Affect Sleep Quality in Hospitalized Patients with COVID-19 Pneumonia. Journal of Turkish Sleep Medicine. 2022;9(1):51-6.

151. Tereshchenko LG, Johnson K, Khayyat-Kholghi M, Johnson B. Rate of Angiotensin-Converting Enzyme Inhibitors and Angiotensin Receptor Blockers Use and the Number of COVID-19-Confirmed Cases and Deaths. AMERICAN JOURNAL OF CARDIOLOGY. 2022;165:101-8.

152. Thomas M. The Fatigue-Related Symptoms Post-Acute SARS-CoV-2: A Preliminary Comparative Study. International Journal of Environmental Research and Public Health. 2022;19(18):11662.

153. Trakada A, Nikolaidis PT, Andrade MS, Puccinelli PJ, Economou NT, Steiropoulos P, et al. Sleep during "lockdown" in the COVID-19 pandemic. International Journal of Environmental Research and Public Health. 2020;17(23):1-10.

154. Trakada A, Nikolaidis PT, Economou NT, Kallianos A, Nena E, Steiropoulos P, et al. Comparison of sleep characteristics during the first and second period of restrictive measures due to COVID-19 pandemic in Greece. European Review for Medical and Pharmacological Sciences. 2022;26(4):1382-7.

155. Tsukahara Y, Hieda Y, Takayanagi S, Macznik A. Risk Factors for Contracting COVID-19 and Changes in Menstrual and Sleep Cycles in Japanese Female Athletes during the COVID-19 Pandemic. Sports (Basel, Switzerland). 2022;10(8).

156. van den Ende ES, van Veldhuizen KDI, Toussaint B, Merten H, van de Ven PM, Kok NA, et al. Hospitalized COVID-19 Patients Were Five Times More Likely to Suffer From Total Sleep Deprivation Compared to Non-COVID-19 Patients; an Observational Comparative Study. Frontiers in Neuroscience. 2021;15:680932.

157. van der Zee-Neuena A, Seymer A, Schaffler-Schaden D, Herfert J, Obrien J, Johansson T, et al. Association of national COVID-19 cases with objectively and subjectively measured mental health proxies in the Austrian Football league - an epidemiological study. ALL LIFE. 2021;14(1):1011-21.

158. Van Duinkerken E, Schmidt GJ, Gjorup ALT, Mello CR, Marques AC, Do Carmo Filho A, et al. Assessment of Attentional Functioning in Health Professionals of a Brazilian Tertiary Referral Hospital for COVID-19. Behavioural Neurology. 2021;2021:6655103.

159. Wang X, Jiang X, Huang Q, Wang H, Gurarie D, Ndeffo-Mbah M, et al. Risk factors of SARS-CoV-2 infection in healthcare workers: a retrospective study of a nosocomial outbreak. Sleep Medicine: X. 2020;2:100028.

160. Weinstock LB, Brook JB, Walters AS, Goris A, Afrin LB, Molderings GJ. Restless legs syndrome is associated with long-COVID in women. JOURNAL OF CLINICAL SLEEP MEDICINE. 2022;18(5):1413-8.

161. Wright L, Steptoe A, Fancourt D. Are adversities and worries during the COVID- 19 pandemic related to sleep quality? Longitudinal analyses of 46,000 UK adults. PLoS ONE. 2021;16(3 March):e0248919.

162. Xiao CX, Lin YJ, Lin RQ, Liu AN, Zhong GQ, Lan CF. Effects of progressive muscle relaxation training on negative emotions and sleep quality in COVID-19 patients: A clinical observational study. Medicine. 2020;99(47):e23185.

163. Yamagami K, Nomura A, Kometani M, Shimojima M, Sakata K, Usui S, et al. Early Detection of Symptom Exacerbation in Patients With SARS-CoV-2 Infection Using the Fitbit Charge 3 (DEXTERITY): Pilot Evaluation. JMIR formative research. 2021;5(9):e30819.

164. Yamamoto JJ, Brandley ET, Ulrich TC. Flight attendant occupational nutrition and lifestyle factors associated with COVID-19 incidence. SCIENTIFIC REPORTS. 2021;11(1).

165. Yang YQ, Liu Y, Pei SL, Yang HH, Wu JJ, Luo CK. Effects of group psychological intervention combined with pulmonary rehabilitation exercises on anxiety and sleep disorders in patients with mild coronavirus disease 2019 (COVID-19) infections in a Fangcang hospital. Psychology, health & medicine. 2022;27(2):333-42.

166. Yildirim G. Evaluation of Early Cognitive Functions in Patients With COVID-19 Infection. The Journal of nervous and mental disease. 2022.

167. Yip T, Feng Y, Fowle J, Fisher CB. Sleep disparities during the COVID-19 pandemic: An investigation of AIAN, Asian, Black, Latinx, and White young adults. Sleep health. 2021;7(4):459-67.

168. Zhang GY, Liu Q, Lin JY, Yan L, Shen L, Si TM. Mental health outcomes among patients from Fangcang shelter hospitals exposed to coronavirus disease 2019: An observational cross-sectional study. Chronic Diseases and Translational Medicine. 2021;7(1):57-64.

169. Zhou FY, Tao MH, Shang LR, Liu YH, Pan GT, Jin Y, et al. Assessment of Sequelae of COVID-19 Nearly 1 Year After Diagnosis. FRONTIERS IN MEDICINE. 2021;8.

170. Zhu Y, Duan MJ, Dijk HH, Freriks RD, Dekker LH, Mierau JO. Association between socioeconomic status and self-reported, tested and diagnosed COVID-19 status during the first wave in the Northern Netherlands: A general population-based cohort from 49 474 adults. BMJ Open. 2021;11(3):e048020.

171. Abbas AM, Kamel MM. Dietary habits in adults during quarantine in the context of COVID-19 pandemic. Obesity medicine. 2020;19:100254.

172. Abokalawa F, Ahmad SF, Al-Hashel J, Hassan AM, Arabi M. The effects of coronavirus disease 2019 (COVID-19) pandemic on people with epilepsy (PwE): an online survey-based study. Acta Neurologica Belgica. 2022;122(1):59-66.

173. Alhayyani RMA, Qassem MY, Alhayyani AMA, Al-Garni AM, Raffaa HS, Al Qarni HZM, et al. Sleep patterns and predictors of poor sleep quality among Saudi commission residents in the Aseer region, Saudi Arabia before and during covid-19 pandemic. J Family Med Prim Care. 2022;11(6):2768-73.

174. Alkodaymi MS, Omrani OA, Fawzy NA, Abou Shaar B, Almamlouk R, Riaz M, et al. Prevalence of post-acute COVID-19 syndrome symptoms at different follow-up periods: a systematic review and meta-analysis. CLINICAL MICROBIOLOGY AND INFECTION. 2022;28(5):657-66.

175. Alyami H, Krageloh CU, Medvedev ON, Alghamdi S, Alyami M, Althagafi J, et al. Investigating Predictors of Psychological Distress for Healthcare Workers in a Major Saudi COVID-19 Center. International Journal of Environmental Research and Public Health. 2022;19(8):4459.

176. Andersen ML, Tufik S. The Association Between Sleep Disturbances and Erectile Dysfunction During the COVID-19 Pandemic. SEXUAL MEDICINE REVIEWS. 2022;10(2):263-70.

177. Atas DB, Sunbul EA, Velioglu A, Tuglular S. The association between perceived stress with sleep quality, insomnia, anxiety and depression in kidney transplant recipients during Covid-19 pandemic. PLoS ONE. 2021;16(3 March):e0248117.

178. Badellino H, Gobbo ME, Torres E, Aschieri ME. Early indicators and risk factors associated with mental health problems during COVID-19 quarantine: Is there a relationship with the number of confirmed cases and deaths? The International journal of social psychiatry. 2021;67(5):567-75.

179. Barnes LA, Leach M, Anheyer D, Brown D, Care J, Lauche R, et al. The effects of Hedera helix on viral respiratory infections in humans: A rapid review. Advances in Integrative Medicine. 2020;7(4):222-6.

180. Barone MTU, Ngongo B, Menna-Barreto L. Sleep-wake cycle impairment adding on the risk for COVID-19 severity in people with diabetes. Sleep Science. 2020;13(3):191-4.

181. Bashir A, Bashir S, Rana K, Lambert P, Vernallis A. Post-COVID-19 Adaptations; the Shifts Towards Online Learning, Hybrid Course Delivery and the Implications for Biosciences Courses in the Higher Education Setting. FRONTIERS IN EDUCATION. 2021;6.

182. Bi K, Chen S. Sleep profiles as a longitudinal predictor for depression magnitude and variability following the onset of COVID-19. Journal of Psychiatric Research. 2022;147:159-65.

183. Borges U, Lobinger B, Javelle F, Watson M, Mosley E, Laborde S. Using Slow-Paced Breathing to Foster Endurance, Well-Being, and Sleep Quality in Athletes During the COVID-19 Pandemic. Frontiers in psychology. 2021;12:624655.

184. Bortolon C, Capdevielle D, Dubreucq J, Raffard S. Persecutory ideation and anomalous perceptual experiences in the context of the COVID-19 outbreak in France: what's left one month later? Journal of Psychiatric Research. 2021;134:215-22.

185. Bougard L, Minguet P, Colson C, Lambermont B, Misset B, Rousseau AF. Post-intensive care syndrome after critical COVID-19 pneumonia. Intensive Care Medicine Experimental. 2021;9(SUPPL 1).

186. Cakir C, Ulus Y, Bilgici A. THE COMPARISON of EXECUTIVE FUNCTIONS of RECOVERED COVID-19 PATIENTS with HEALTHY CONTROLS. Annals of the Rheumatic Diseases. 2022;81(Supplement 1):1703-4.

187. Caroppo E, Mazza M, Sannella A, Marano G, Avallone C, Claro AE, et al. Will Nothing Be the Same Again?: Changes in Lifestyle during COVID-19 Pandemic and Consequences on Mental Health. INTERNATIONAL JOURNAL OF ENVIRONMENTAL RESEARCH AND PUBLIC HEALTH. 2021;18(16).

188. Ciavarella D, Tepedino M, Troiano G. State of the Art in Comorbidities and Complications Associated with Sleep-Related Breathing Disorders. APPLIED SCIENCES-BASEL. 2021;11(17).

189. Cunha TCA, Cunha TM, Matoso AGB, Januzzi E, Dal-Fabbro C. COVID-19 - The clinical consequences of social isolation and the relation with sleep bruxism and comorbidities. Sleep Science. 2021;14(4):366-9.

190. Cunha TCA, Rossi R, Marson LCG, Guimaraes TM, MacHado MAC, Meira ECM, et al. COVID-19 and dental sleep medicine: Risks, precautions and patient guidance. Sleep Science. 2020;13(3):195-8.

191. Datta K, Tripathi M. Sleep and Covid-19. Neurology India. 2021;69(1):26-31.

192. de Almondes KM, Castro EAS, Paiva T. Morbidities Worsening Index to Sleep in the Older Adults During COVID-19: Potential Moderators. Frontiers in psychology. 2022;13:913644.

193. De Mello MT, Silva A, Guerreiro RD, da-Silva FR, Esteves AM, Poyares D, et al. Sleep and COVID-19: considerations about immunity, pathophysiology, and treatment. SLEEP SCIENCE. 2020;13(3):199-209.

194. de Sousa Martins e Silva E, Ono BHVS, Souza JC. Sleep and immunity in times of COVID-19. Revista da Associacao Medica Brasileira. 2020;66:143-7.

195. Desdiani D, Sutarto AP. Impact of the restrictions on community activities policy during the COVID-19 on psychological health in Indonesia's urban and rural residents: A cross-sectional study. Health Science Reports. 2022;5(5):e725.

196. Dimitrievski A, Zdravevski E, Lameski P, Villasana MV, Miguel Pires I, Garcia NM, et al. Towards Detecting Pneumonia Progression in COVID-19 Patients by Monitoring Sleep Disturbance Using Data Streams of Non-Invasive Sensor Networks. Sensors (Basel, Switzerland). 2021;21(9).

197. Dos Santos ACV, Landin NNDS, Martins LC, Nunes CTG, Silveira SS, Da Silva MMC, et al. Sleep quality in patients in the rehabilitation phase after COVID-19 diagnosis. Sleep Science. 2022;15(Supplement 3):76.

198. Dutta S, Kaur R, Charan J, Bhardwaj P, Ambwani SR, Babu S, et al. Analysis of Neurological Adverse Events Reported in VigiBase From COVID-19 Vaccines. Cureus. 2022;14(1):e21376.

199. Eldringhoff H, Mickelson C, Moore L, Pirner M, Doyle S, Mantua J, et al. An analysis of objective and subjective sleep and infection symptoms of medical personnel working through the COVID-19 pandemic. Sleep. 2021;44(SUPPL 2):A269-A70.

200. Frange C, de Oliveira GP, Coelho FMS. COVID-19 and Central Nervous System Hypersomnias. Current Sleep Medicine Reports. 2022;8(3):42-9.

201. Fu L, Fang Y, Luo D, Wang B, Xiao X, Hu Y, et al. Erratum: Pre-hospital, in-hospital and post-hospital factors associated with sleep quality among COVID-19 survivors 6 months after hospital discharge: Cross-sectional survey in five cities in China (BJPsych Open (2021) 7:6 (E191) DOI: 10.1192/bjo.2021.1008). BJPsych Open. 2021;7(6):e214.

202. Gao C, Scullin MK. Sleep health early in the coronavirus disease 2019 (COVID-19) outbreak in the United States: integrating longitudinal, cross-sectional, and retrospective recall data. Sleep Medicine. 2020;73:1-10.

203. Ghelbash Z, Rad MN. Evaluation of sleep quality and its related factors in the elderly with a history of covid-19. International Journal of Pharmaceutical Research. 2021;13(1):6003-8.

204. Gokmen K, Atas DB, Tugcu M, Velioglu A, Arikan IH, Alibaz-Oner F, et al. THE RELATIONSHIP BETWEEN PERCEIVED STRESS WITH ANXIETY, DEPRESSION, SLEEP QUALITY, INSOMNIA AND DRUG ADHERENCE IN PATIENTS WITH SYSTEMIC LUPUS ERYTHEMATOSUS DURING THE COVID-19 PANDEMIC. Nephrology Dialysis Transplantation. 2022;37(SUPPL 3):i70-i1.

205. Guo Y, Cheng C, Zeng Y, Li Y, Zhu M, Yang W, et al. Mental health disorders and associated risk factors in quarantined adults during the COVID-19 outbreak in China: Cross-sectional study. Journal of Medical Internet Research. 2020;22(8):20328.

206. Guzenko D, Garcia G, Siyahjani F, Monette K, DeFranco S, Sikri D, et al. Longitudinal, unobtrusive, and ecologically valid sleep metric estimation from a smart bed to predict the pathology of COVID-19. Sleep. 2021;44(SUPPL 2):A255.

207. Hackett B, Badami V, Sharma S, Stansbury R. Sleep quality after COVID-19 infection. Sleep. 2021;44(SUPPL 2):A272-A3.

208. Hailu Tesfaye A, Alemayehu M, Abere G, Kabito GG. Risk factors for the prevalence of poor sleep quality in lecturers during COVID-19 pandemic in Ethiopia: an institution-based cross-sectional study. BMJ open. 2022;12(10):e066024.

209. Harriger JA, Joseph NT, Trammell J. Detrimental Associations of Cumulative Trauma, COVID-19 Infection Indicators, Avoidance, and Loneliness With Sleep and Negative Emotionality in Emerging Adulthood During the Pandemic. EMERGING ADULTHOOD. 2021;9(5):479-91.

210. Helm EE, Kempski KA, Galantino MLA. Effect of disrupted rehabilitation services on distress and quality of life in breast cancer survivors during the COVID-19 pandemic. Rehabilitation Oncology. 2020;38(4):153-8.

211. Hetkamp M, Schweda A, Bauerle A, Weismuller B, Kohler H, Musche V, et al. Sleep disturbances, fear, and generalized anxiety during the COVID-19 shut down phase in Germany: relation to infection rates, deaths, and German stock index DAX. Sleep Medicine. 2020;75:350-3.

212. Invitto S, Romano D, Garbarini F, Bruno V, Urgesi C, Curcio G, et al. Major Stress-Related Symptoms During the Lockdown: A Study by the Italian Society of Psychophysiology and Cognitive Neuroscience. Frontiers in public health. 2021;9:636089.

213. Jagannathan S, Rodgers M, McCrae CS, Miller MB, Curtis A. COVID-19 anxiety and sleep in middle-aged and older adults: Impact of age and sex. Sleep. 2021;44(SUPPL 2):A262-A3.

214. Jangalee JV, Ghasvareh P, Guenette JA, Road J. Incorporating remote patient monitoring in virtual pulmonary rehabilitation programs. Canadian Journal of Respiratory Therapy. 2021;57:83-9.

215. Kamp K, Murphy T, Shulman RJ, Van Tilburg MA, Romano JM, Levy RL. IMPACT OF THE COVID-19 PANDEMIC ON ABDOMINAL PAIN, EMOTIONAL DISTRESS, QUALITY OF LIFE, SLEEP, AND DISABILITY IN CHILDREN WITH FUNCTIONAL ABDOMINAL PAIN DISORDERS. GASTROENTEROLOGY. 2022;162(7):S57-S8.

216. Karkala A, Moschonas S, Sykas G, Karagianni M, Gilou S, Papaefthymiou O, et al. Sleep Quality and Mental Health Consequences of COVID-19 Pandemic in the Aviation Community in Greece. Journal of Occupational and Environmental Medicine. 2022;64(9):E567-E74.

217. Kim SW, Su KP. Using psychoneuroimmunity against COVID-19. Brain, Behavior, and Immunity. 2020;87:4-5.

218. Lemoine P, Ebert D, Koga Y, Bertin C. Public interest and awareness regarding general health, sleep quality and mental wellbeing during the early COVID-19 pandemic period: An exploration using Google trends. Sleep epidemiology. 2022;2:100017.

219. Li Y, Miller M, Torrence N. A novel modularized intervention to improve sleep in older hospitalized veterans. Sleep. 2021;44(SUPPL 2):A294.

220. Liskova A, Koklesova L, Samec M, Abdellatif B, Zhai K, Siddiqui M, et al. Targeting phytoprotection in the COVID-19-induced lung damage and associated systemic effects-the evidence-based 3PM proposition to mitigate individual risks. EPMA Journal. 2021;12(3):325-47.

221. Lucchini M, Firestein M, Shuffrey LC, Pini N, Babineau V, Fifer WP, et al. Mental health clusters during COVID-19 pandemic are associated with multiple dimensions of sleep in a sample of pregnant women. Sleep. 2021;44(SUPPL 2):A75-A6.

222. Lucchini M, Kyle MH, Sania A, Pini N, Babineau V, Firestein MR, et al. Postpartum sleep health in a multiethnic cohort of women during the COVID-19 pandemic in New York City. Sleep health. 2022;8(2):175-82.

223. Magnavita N, Soave PM, Antonelli M. Prolonged stress causes depression in frontline workers facing the covid-19 pandemic-a repeated cross-sectional study in a covid-19 hub-hospital in central italy. International Journal of Environmental Research and Public Health. 2021;18(14):7316.

224. Malik A, Arif M, Syed NA, Bashir MK, Ali N. Epidemiology of sleeping disorders among doctors during COVID-19 pandemic in Pakistan. Pakistan Journal of Medical and Health Sciences. 2022;16(4):592-3.

225. Mantovani E, Mariotto S, Gabbiani D, Dorelli G, Bozzetti S, Federico A, et al. Chronic fatigue syndrome: an emerging sequela in COVID-19 survivors? Journal of NeuroVirology. 2021;27(4):631-7.

226. Marmet S, Wicki M, Gmel G, Gachoud C, Daeppen JB, Bertholet N, et al. The psychological impact of the COVID-19 crisis on young Swiss men participating in a cohort study. Swiss Medical Weekly. 2021;151(39-40):w30028.

227. Mayer KP, Sturgill JL, Kalema AG, Soper MK, Seif SM, Cassity EP, et al. Recovery from COVID-19 and acute respiratory distress syndrome: the potential role of an intensive care unit recovery clinic: a case report. JOURNAL OF MEDICAL CASE REPORTS. 2020;14(1).

228. Mo GH, Wang ZX, Chen XS, Jiang Q. The prognosis and prevention measures for mental health in COVID-19 patients: through the experience of SARS. BioPsychoSocial medicine. 2020;14:22.

229. Mollerup A, Larsen SC, Bennetzen AS, Henriksen M, Simonsen MK, Weis N, et al. PEP-CoV protocol: A PEP flute-self-care randomised controlled trial to prevent respiratory deterioration and hospitalisation in early COVID-19. BMJ Open. 2021;11(6):e050582.

230. Munoz-Ceron J, Gallo L, Suarez J. Clinical Course of Migraine during Strict Quarantine due to SARS-CoV-2: Effect of Psychiatric Comorbidities in a Clinical Cohort. European Neurology. 2021;84(5):348-53.

231. Murga I, Aranburu L, Gargiulo PA, Gomez Esteban JC, Lafuente JV. Clinical Heterogeneity in ME/CFS. A Way to Understand Long-COVID19 Fatigue. Frontiers in Psychiatry. 2021;12:735784.

232. Neculicioiu VS, Colosi IA, Costache C, Sevastre-Berghian A, Clichici S. A Review of the Impact of the COVID-19 Pandemic on Sleep and Mental Health. International Journal of Environmental Research and Public Health. 2022;19(6):3497.

233. Nguyen N, Mebust K. New-onset obstructive sleep apnea diagnosis in a COVID-positive patient. Sleep. 2021;44(SUPPL 2):A336.

234. O'Regan D, Jackson ML, Young AH, Rosenzweig I. Understanding the Impact of the COVID-19 Pandemic, Lockdowns and Social Isolation on Sleep Quality. Nature and science of sleep. 2021;13:2053-64.

235. O'Sullivan SE, Stevenson CW, Laviolette SR. Could Cannabidiol Be a Treatment for Coronavirus Disease-19-Related Anxiety Disorders? CANNABIS AND CANNABINOID RESEARCH. 2021;6(1):7-18.

236. Pan Y, Xin M, Zhang C, Dong W, Fang Y, Wu W, et al. Associations of mental health and personal preventive measure compliance with exposure to COVID-19 information during work resumption following the COVID-19 outbreak in China: Cross-sectional survey study. Journal of Medical Internet Research. 2020;22(10):e22596.

237. Patience M, Burns RE, Cassidy D. Adjusting fire-piloting an online sleep class in a U.S. air force population in response to COVID-19 patient care restrictions. Sleep. 2021;44(SUPPL 2):A257.

238. Pitera P, Gobbi M, Fontana JM, Cattaldo S, Massucci M, Capodaglio P. Whole-Body Cryostimulation: A Rehabilitation Booster in Post-COVID Patients? A Case Series. APPLIED SCIENCES-BASEL. 2022;12(10).

239. Ran L, Tan XD. Working Schedule, Sleep Quality, and Susceptibility to Coronavirus Disease 2019 in Healthcare Workers Reply. CLINICAL INFECTIOUS DISEASES. 2021;72(9):1676-7.

240. Rogers A, Blanc J, Seixas A, Nunes J, Casimir G, Jean-Louis G. Sleep latency, pre and peri-COVID-19 experiences and PTSD symptoms: Results from the NYU COVID-19 mental health study. Sleep. 2021;44(SUPPL 2):A267-A8.

241. Saeed SA, Pastis IS, Santos MG. COVID-19 and its impact on the brain and Mind- A conceptual model and supporting evidence. Psychiatric Quarterly. 2022;93(1):271-84.

242. Sargin K. DETERMINATION OF FOOTBALLERS ANXIETY AND SLEEP QUALITY OF GETTING INFECTED BY NEW TYPE CORONAVIRUS. INTERNATIONAL JOURNAL OF LIFE SCIENCE AND PHARMA RESEARCH. 2021:88-93.

243. Semyachkina-Glushkovskaya O, Mamedova A, Vinnik V, Klimova M, Saranceva E, Ageev V, et al. Brain Mechanisms of COVID-19-Sleep Disorders. INTERNATIONAL JOURNAL OF MOLECULAR SCIENCES. 2021;22(13).

244. Sethi Y, Kaiwan O, Bassiony M, Vora V, Agarwal P, Gajwani N, et al. Psychological Assessment of Family Caregivers of Patients With COVID-19 in the United States of America and India. Cureus. 2022;14(9):e29267.

245. Shah PJ, Ghodge SV. Effect of guided imagery as an adjunct to pulmonary rehabilitation on anxiety, quality of life, and quality of sleep in a post COVID-19 patient via telerehabilitation. JOURNAL OF MENTAL HEALTH AND HUMAN BEHAVIOUR. 2021;26(2):166-8.

246. Singh RB, Juneja L, Wilson DW, De Meester F, Wilczynska A, Takahashi T, et al. Diet and Lifestyle Guidelines for Immunomodulation with Reference to Corona Virus Pandemic: A Scientific Statement of the International College of Nutrition. World Heart Journal. 2021;13(4):499-517.

247. Smirmaul BPC, Chamon RF, de Moraes FM, Rozin G, Moreira ASB, de Almeida R, et al. Lifestyle Medicine During (and After) the COVID-19 Pandemic. AMERICAN JOURNAL OF LIFESTYLE MEDICINE. 2021;15(1):60-7.

248. Tasdemir Yigitoglu G, Yilmaz A, Yilmaz H. The effect of Covid-19 on sleep quality, anxiety and depression on healthcare staff at a tertiary hospital in Turkey. Archives of psychiatric nursing. 2021;35(5):504-10.

249. Udeh-Momoh CT, Watermeyer T, Sindi S, Giannakopoulou P, Robb CE, Ahmadi-Abhari S, et al. Health, Lifestyle, and Psycho-Social Determinants of Poor Sleep Quality During the Early Phase of the COVID-19 Pandemic: A Focus on UK Older Adults Deemed Clinically Extremely Vulnerable. Frontiers in public health. 2021;9:753964.

250. Ustundag G, Ozyurt G, Kara Aksay A, Sahin A, Ekemen Keles Y, Ozturk Y, et al. Evaluation of Depression, Anxiety, and Sleep Quality in Children Diagnosed with COVID-19. Journal of Nervous and Mental Disease. 2022;210(8):629-32.

251. Vicente BM, Neto JV, Quaresma M, Vasconcelos JS, Espíndola Bauchiunas R, Dos Santos ECM, et al. Covid-19 Social Distancing, Lifestyle and Health Outcomes Among Persons Living with HIV (PLWH): A Web-based Survey. AIDS and behavior. 2022:1-12.

252. Viehmann-Wical K. Evaluating sleep quality with coronavirus disease 2019 (COVID-19) testing. Journal of Clinical Sleep Medicine. 2021;17(2):355.

253. Vitale JA, Perazzo P, Silingardi M, Biffi M, Banfi G, Negrini F. Is disruption of sleep quality a consequence of severe Covid-19 infection? A case-series examination. Chronobiology international. 2020;37(7):1110-4.

254. Wagner-Skacel J, Dalkner N, Bengesser S, Ratzenhofer M, Fink N, Kahn J, et al. COVID-19 Pandemic Stress-Induced Somatization in Transplant Waiting List Patients. Frontiers in Psychiatry. 2021;12:671383.

255. Wagner-Skacel J, Dalkner N, Ratzenhofer M, Fink N, Kahn J, Kniepeiss D, et al. Covid-19 pandemic induces stress-induced somatization in wait list patients. Transplant International. 2020;33(SUPPL 2):17.

256. Wong AW, Shah AS, Hague CJ, Johnston JC, Ryerson CJ, Carlsten C. Natural history of COVID-19 recovery: Changes in physiologic, radiologic and patient-reported outcomes 12 months after symptom onset. CANADIAN JOURNAL OF RESPIRATORY CRITICAL CARE AND SLEEP MEDICINE. 2022;6(4):270-4.

257. Yoka O, Yoka K, Turan MB, Guler E. The relationship between individual and team athletes' anxiety to catch the novel coronavirus (covid-19) and sleep qualitay. VIREF-REVISTA DE EDUCACION FISICA. 2022;11(1):114-33.

258. Zambrelli E, Canevini M, Gambini O, D'Agostino A. Delirium and sleep disturbances in COVID–19: A possible role for melatonin in hospitalized patients? Sleep Medicine. 2020;70:111-.

259. Zarei S, Fooladvand K. Mediating effect of sleep disturbance and rumination on work-related burnout of nurses treating patients with coronavirus disease. BMC psychology. 2022;10(1):197.

260. Zhang C, Yang L, Liu S, Ma S, Wang Y, Cai Z, et al. Survey of Insomnia and Related Social Psychological Factors Among Medical Staff Involved in the 2019 Novel Coronavirus Disease Outbreak. Front Psychiatry. 2020;11:306.

261. Zhang R, Wang X, Ni L, Di X, Ma B, Niu S, et al. COVID-19: Melatonin as a potential adjuvant treatment. Life Sciences. 2020;250:117583.

262. Zhao X, Zhang T, Li B, Yu X, Ma Z, Cao L, et al. Job-related factors associated with changes in sleep quality among healthcare workers screening for 2019 novel coronavirus infection: a longitudinal study. Sleep Medicine. 2020;75:21-6.

263. Zhu MC, Chen DL, Zhu Y, Xiong XS, Ding Y, Guo FB, et al. Long-term sero-positivity for IgG, sequelae of respiratory symptoms, and abundance of malformed sperms in a patient recovered from severe COVID-19. EUROPEAN JOURNAL OF CLINICAL MICROBIOLOGY & INFECTIOUS DISEASES. 2021;40(7):1559-67.

264. Zsuffa JA, Koszovácz V, Berente DB, Bálint Z, Katz S, Kamondi A, et al. A COVID-19-pandémia harmadik hullámának hatása a 60 év feletti magyar lakosság életmódjára, mentális és fizikai egészségére. Orvosi hetilap. 2022;163(31):1215-23.

265. Samushiya MA, Kryzhanovsky SM, Ragimova AA, Berishvili TZ, Chorbinskaya SA, Ivannikova EI. Psychoemotional Disorders and Sleep Impairments in Patients with COVID-19. Neuroscience and Behavioral Physiology. 2022;52(2):231-5.

266. Fernandez-de-las-Penas C, Gomez-Mayordomo V, Cuadrado ML, Palacios-Cena D, Florencio LL, Guerrero AL, et al. The presence of headache at onset in SARS-CoV-2 infection is associated with long-term post-COVID headache and fatigue: A case-control study. Cephalalgia. 2021;41(13):1332-41.

267. Fernandez-de-las-Penas C, Gomez-Mayordomo V, Garcia-Azorin D, Palacios-Cena D, Florencio LL, Guerrero AL, et al. Previous History of Migraine Is Associated With Fatigue, but Not Headache, as Long-Term Post-COVID Symptom After Severe Acute Respiratory SARS-CoV-2 Infection: A Case-Control Study. Frontiers in Human Neuroscience. 2021;15:678472.

268. Fernandez-de-Las-Penas C, Martin-Guerrero JD, Cancela-Cilleruelo I, Moro-Lopez-Menchero P, Rodriguez-Jimenez J, Pellicer-Valero OJ. Trajectory Curves of post-COVID Anxiety/Depressive Symptoms and Sleep Quality in Previously Hospitalized COVID-19 Survivors: The LONG-COVID-EXP-CM Multicenter Study. Psychological medicine. 2022:1-8.

269. Fernandez-De-las-penas C, Martin-Guerrero JD, Pellicer-Valero OJ, Navarro-Pardo E, Gomez-Mayordomo V, Cuadrado ML, et al. Female Sex Is a Risk Factor Associated with Long-Term Post-COVID Related-Symptoms but Not with COVID-19 Symptoms: The LONG-COVID-EXP-CM Multicenter Study. Journal of Clinical Medicine. 2022;11(2):413.

270. Fernandez-de-las-Penas C, Ortega-Santiago R, Fuensalida-Novo S, Martin-Guerrero JD, Pellicer-Valero OJ, Torres-Macho J. Differences in Long-COVID Symptoms between Vaccinated and Non-Vaccinated (BNT162b2 Vaccine) Hospitalized COVID-19 Survivors Infected with the Delta Variant. Vaccines. 2022;10(9):1481.

271. Fernandez-de-Las-Penas C, Rodriguez-Jimenez J, Fuensalida-Novo S, Palacios-Cena M, Gomez-Mayordomo V, Florencio LL, et al. Myalgia as a symptom at hospital admission by severe acute respiratory syndrome coronavirus 2 infection is associated with persistent musculoskeletal pain as long-term post-COVID sequelae: a case-control study. Pain. 2021;162(12):2832-40.

272. Fernandez-de-las-Penas C, Torres-Macho J, Elvira-Martinez CM, Molina-Trigueros LJ, Sebastian-Viana T, Hernandez-Barrera V. Obesity is associated with a greater number of long-term post-COVID symptoms and poor sleep quality: A multicentre case-control study. International Journal of Clinical Practice. 2021;75(12):e14917.

273. Fernoandez-De-las-Penas C, Guijarro C, Torres-Macho J, Velasco-Arribas M, Plaza-Canteli S, Hernoandez-Barrera V, et al. Diabetes and the Risk of Long-term Post-COVID Symptoms. Diabetes. 2021;70(12):2917-21.

274. Kabir KS, Flis A, Mickens M, Trapp SK, Wiese J. "We're Not Meant to Deal with Crisis for a Year": Supporting Frontline Healthcare Providers' Wellness During a Pandemic. PERVASIVE COMPUTING TECHNOLOGIES FOR HEALTHCARE, PERVASIVE HEALTH 20212022. p. 147-63.

275. Pataka A, Kotoulas S, Sakka E, Katsaounou P, Pappa S. Sleep Dysfunction in COVID-19 Patients: Prevalence, Risk Factors, Mechanisms, and Management. JOURNAL OF PERSONALIZED MEDICINE. 2021;11(11).

276. Satashia P, Castellanos P, Aleger C, Yin M, Cheung J. SLEEP DISTURBANCE AMONG COVID-19 POSTHOSPITALIZED PATIENTS. Sleep. 2022;45(SUPPL 1):A247.

277. Bellou V, Tzoulaki I, van Smeden M, Moons KGM, Evangelou E, Belbasis L. Prognostic factors for adverse outcomes in patients with COVID-19: a field-wide systematic review and meta-analysis. EUROPEAN RESPIRATORY JOURNAL. 2022;59(2).

278. Garbarina S. Neurophysiology of sleep in workers' health and safety during Covid19 pandemic. Safety and Health at Work. 2022;13(Supplement):S18.

279. Vitalakumar D, Sharma A, Kumar A, Flora SJS. Neurological Manifestations in COVID-19 Patients: A Meta-Analysis. ACS CHEMICAL NEUROSCIENCE. 2021;12(15):2776-97.

280. Qian YL, Xu H, Diao J, Li QZ, Zhan Q, Fang YJ. Influence of life intervention on anxiety, depression, and quality of life of COVID-19 patients A protocol for systematic review and meta-analysis. MEDICINE. 2021;100(18).

281. Schmitz NCM, van der Werf YD, Lammers-van der Holst HM. The Importance of Sleep and Circadian Rhythms for Vaccination Success and Susceptibility to Viral Infections. Clocks & sleep. 2022;4(1):66-79.

282. Sirbu E, Gligor S, Gligor R, Ianc D. Evaluation of Health-Related Quality of Life in Rheumatoid Arthritis Patients During the COVID-19 Pandemic. PROCEEDINGS OF THE 6TH INTERNATIONAL CONFERENCE OF UNIVERSITARIA CONSORTIUM FEFSTIM: PHYSICAL EDUCATION, SPORTS AND KINESIOTHERAPY - IMPLICATIONS IN QUALITY OF LIFE2020. p. 336-42.

283. Ding H, He F, Lu YG, Hao SW, Fan XJ. Effects of non-drug interventions on depression, anxiety and sleep in COVID-19 patients: a systematic review and meta-analysis. EUROPEAN REVIEW FOR MEDICAL AND PHARMACOLOGICAL SCIENCES. 2021;25(2):1087-96.

284. Magnusdottir I, Lovik A, Unnarsdottir AB, McCartney D, Ask H, Koiv K, et al. Acute COVID-19 severity and mental health morbidity trajectories in patient populations of six nations: an observational study. The Lancet Public health. 2022;7(5):e406-e16.

285. Parodi C, Vigano I, Ottaviano E, Massa V, Borghi E, Beretta S, et al. Long-term analysis of the effects of COVID-19 in people with epilepsy: Results from a multicenter on-line survey across the pandemic waves. Epilepsy and Behavior. 2022;135:108900.

286. Pellitteri G, Surcinelli A, De Martino M, Fabris M, Janes F, Bax F, et al. Sleep alterations following COVID-19 are associated with both neuroinflammation and psychological disorders, although at different times. Frontiers in Neurology. 2022;13:929480.

287. Salfi F, D'Atri A, Tempesta D, Ferrara M. Sleeping under the waves: A longitudinal study across the contagion peaks of the COVID-19 pandemic in Italy. Journal of Sleep Research. 2021;30(5):e13313.

288. Stavrou VT, Tourlakopoulos KN, Vavougios GD, Papayianni E, Kiribesi K, Maggoutas S, et al. Eight weeks unsupervised pulmonary rehabilitation in previously hospitalized of sars-cov-2 infection. Journal of Personalized Medicine. 2021;11(8):806.

289. Wong AW, Shah AS, Hague CJ, Johnston JC, Ryerson CJ, Carlsten C. The Natural History of COVID-19 Recovery: Changes in Physiologic, Radiologic, and Patient Reported Outcomes 12 Months After Symptom Onset. American Journal of Respiratory and Critical Care Medicine. 2022;205(1).

290. Yang X, Kumar P, Cao B, Ma X, Li T. Social support and clinical improvement in COVID-19 positive patients in China. Nursing outlook. 2020;68(6):830-7.

291. Yilmaz Kafali H, Turan S, Akpinar S, Mutlu M, Ozkaya Parlakay A, Cop E, et al. Correlates of psychotic like experiences (PLEs) during Pandemic: An online study investigating a possible link between the SARS-CoV-2 infection and PLEs among adolescents. Schizophrenia Research. 2022;241:36-43.

292. Zhang L, Li T, Chen L, Wu F, Xia W, Huang M, et al. Association of sleep quality before and after sars-cov-2 infection with clinical outcomes in hospitalized patients with covid-19 in China. EXCLI Journal. 2021;20:894-906.
